# Tissue‐Resident Macrophage‐Derived E3 Ligase SMURF2 Restricts Autoimmune Inflammation by Mediating the Degradation of p‐TBK1

**DOI:** 10.1002/advs.202504930

**Published:** 2025-11-21

**Authors:** Xiang An, Jun Li, Lingling Wang, Chengyuan Li, Zhenpeng Jin, Yushi Yao, Minmin Jiang, Wenlong Lin, Xiaojian Wang

**Affiliations:** ^1^ Institute of Immunology and Bone Marrow Transplantation Center of The First Affiliated Hospital School of Medicine Zhejiang University Hangzhou 310058 China; ^2^ Liangzhu Laboratory Zhejiang University Medical Center 1369 West Wenyi Road Hangzhou 311121 China; ^3^ Department of Pathology the First Affiliated Hospital School of Medicine Zhejiang University Hangzhou 310003 China; ^4^ Shulan International Medical College Zhejiang Shuren University Hangzhou 310015 China; ^5^ The Second Affiliated Hospital Zhejiang University School of Medicine Hangzhou China

**Keywords:** autoimmune diseases, inflammatory bowel disease, p‐TBK1, tissue‐resident macrophages, ubiquitination

## Abstract

Dysregulated tissue‐resident macrophages (TRMs) contribute to the pathogenesis of inflammatory bowel disease (IBD) and multiple sclerosis (MS). Uncovering molecular regulators of the divergent role of TRMs in inflammation can advance therapeutic strategies for autoimmune disorders. Here, a significant downregulation of SMAD‐specific E3 ubiquitin protein ligase 2 (SMURF2) is reported in TRMs within inflamed intestinal tissues from both IBD patients and mouse models. Notably, TRM‐specific deficiency of *Smurf2* significantly exacerbates TRM proliferation in dextran sulfate sodium (DSS)‐induced colitis and experimental autoimmune encephalomyelitis (EAE), leading to augmented autoimmune inflammation. Mechanistically, SMURF2 interacts with phosphorylated TBK1 (p‐TBK1), mediating its Lys‐27‐linked ubiquitination and its subsequent lysosomal degradation, thereby suppressing TRM proliferation and autoimmune inflammation. Collectively, these findings establish SMURF2 as a pivotal mediator of TRM proliferation and autoimmune inflammation via p‐TBK1 modulation. Given that impaired SMURF2 expression correlates with the progression of autoimmune inflammation, SMURF2 represents a potential target for autoimmune disease treatment.

## Graphical abstract

Dysregulated Tissue resident macrophage (TRMs) link to autoimmune inflammation. SMURF2 mediates Lys‐27 (K27)‐linked ubiquitination of p‐TBK1 and its degradation, which inhibits CSF1R signaling‐triggered TRM proliferation, thereby restraining the autoimmune inflammation. Impaired expression of SMURF2 in TRM correlates with the progression of autoimmune disease in humans and mice.

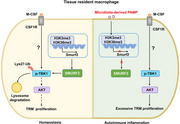

## 1 Introduction

Tissue‐resident macrophages (TRMs) are localized within mucosal tissues, including the intestine, skin, lung, as well as parenchymal organs such as the heart, brain, liver, and spleen.^[^ ^1^ ^]^ TRMs integrate signals from various environmental sensors to orchestrate adaptive cellular responses that are critical for the growth, remodeling, and homeostasis of specialized tissues.^[^ ^2^ ^]^ The differentiation, proliferation, and survival of TRMs are primarily governed by macrophage colony‐stimulating factor (M‐CSF).^[^ ^1^ ^]^ However, as local immune monitors, TRMs have attracted increasing attention for their pro‐inflammatory effects during the early stage of inflammation, particularly in the colon and central nervous system (CNS).^[^ ^3^, ^4^, ^5^ ^]^ Dysregulation of TRMs disrupts their homeostatic functions, causing an elevation in inflammatory mediators and the recruitment of immune cells.Consequently, this leads to tissue damage and accelerates the progression of autoimmune diseases.^[^ ^3^, ^4^ ^]^

Colonic TRMs, identified as CX3CR1^hi^ cells in CD11b^+^ intestinal mononuclear phagocytes,^[^ ^6^ ^]^ distribute throughout the intestinal mucosa and perform crucial functions, particularly in the removal of debris or apoptotic cells.^[^ ^7^ ^]^ In inflammatory environments, dysregulated colonic TRMs release excessive chemokines to recruit Ly6C^+^ monocytes, which differentiate into CX3CR1^int^ pro‐inflammatory monocyte‐derived macrophages (MDMs).MDMs express high levels of proinflammatory mediators and boost a severe inflammatory response.^[^ ^4^, ^8^ ^]^ It has been reported that FBXW7 regulates the CCL2/7 expression in TRMs to promote intestinal inflammation.^[^ ^4^ ^]^

As the resident macrophages of the central nervous system (CNS), microglia derive from the yolk sac and are maintained through self‐renewal within the CNS.^[^ ^9^ ^]^ Microglia are crucial for the pathogenesis of multiple sclerosis (MS) and its animal model, experimental autoimmune encephalomyelitis (EAE).^[^ ^9^ ^]^ During EAE progression, microglia undergo significant proliferation and secrete chemokines and proinflammatory cytokines, which mediate leukocyte recruitment into the CNS and promote inflammation.^[^ ^10^ ^]^ Meanwhile, microglia regulate the death of neurons and oligodendrocytes in neurodegenerative disorders by inducing neurotoxic reactive astrocytes.^[^ ^11^ ^]^ Targeting microglia is an emerging strategy for treating multiple sclerosis.Microglia depletion with CSF‐1R‐specific inhibitor PLX5622 alleviated the development of EAE.^[^ ^12^ ^]^ Consistently, M‐CSF signaling inhibition reduces microglial proliferation and attenuates disease progression in MS.^[^ ^13^ ^]^ Therefore, the overactive immune response in TRMs leads to an imbalance in local homeostasis and triggers autoimmune inflammation in both the colon and the CNS, as seen in inflammatory bowel disease (IBD) and MS.^[^ ^4^, ^14^ ^]^ Clarifying the underlying regulatory mechanisms of TRMs in the progression of autoimmune inflammation will facilitate the development of new therapeutic targets for autoimmune diseases.

SMAD‐specific E3 ubiquitin protein ligase 2 (SMURF2), a highly conserved HECT‐class E3 ligase within the NEDD4 family, functions as a tumor suppressor gene by regulating many key functional proteins, including SATB1, RNF20, YY1.^[^ ^15^ ^]^ It has been reported that macrophage SMURF2 regulates bone homeostasis and suppresses the antiviral immune response by degrading SMAD3^[^ ^16^ ^]^ and MAVS,^[^ ^17^ ^]^ respectively. We recently reported NEDD4L, another NEDD4 family member, mediated intestinal epithelial cell ferroptosis to restrict IBD.^[^ ^18^ ^]^ However, the precise function of SMURF2 in autoimmune diseases remains largely unexplored.

In this study, we identified that SMURF2 restrained the proliferation of TRMs to prevent autoimmune inflammation. *Smurf2* deficiency in TRMs exacerbates the inflammation and disease severity in the colitis and EAE models. Mechanistically, SMURF2 promotes the degradation of phosphorylated Tank binding kinase 1 (p‐TBK1), thereby restricting the proliferation of TRMs. Moreover, the expression of SMURF2 was markedly decreased in inflamed intestinal tissue from IBD patients and mouse models. Collectively, our findings provide insight into the physiological role of SMURF2 in autoimmune disease and propose SMURF2 as a target for preventing autoimmune diseases.

## 2 Results

### 2.1 Reduced SMURF2 Expression in Colonic Macrophages is Associated with IBD Progression

**Figure 1:**
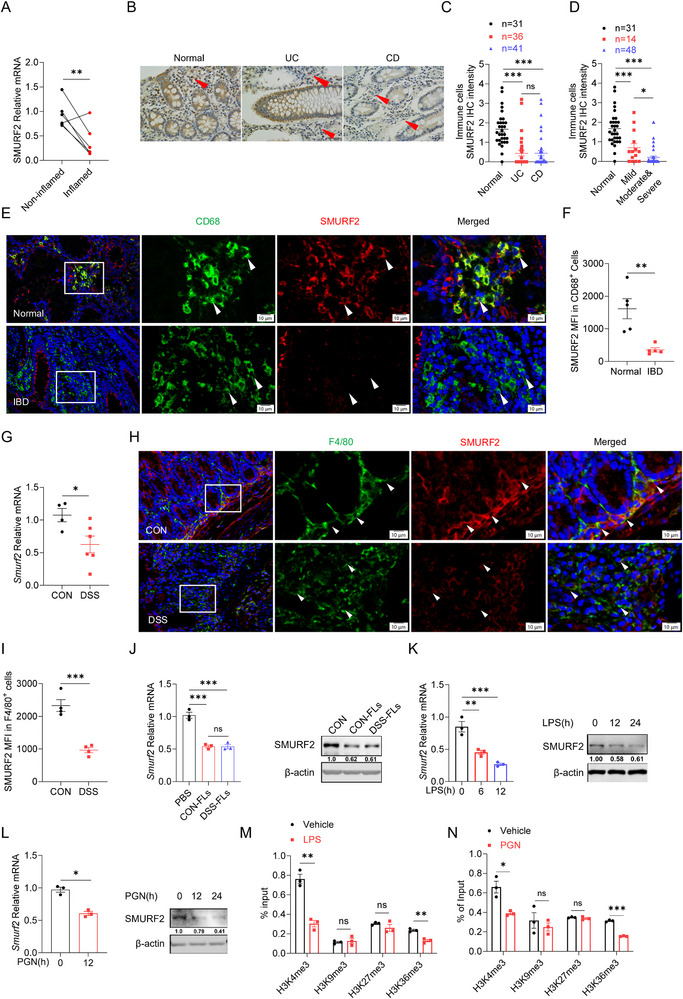
Reduced SMURF2 expression in inflamed intestinal tissues. A) RT‐qPCR analysis of *SMURF2* mRNA expression in paired inflamed and non‐inflamed colon tissue from IBD patients (n = 5). B)Representative SMURF2 immunohistochemistry (IHC) staining of colon specimens obtained from patients with UC or CD and healthy controls. Red arrows indicate SMURF2 expression in immune cells. Scale bars, 100 µm. C,D) Statistical analysis of SMURF2 IHC intensity within mesenchymal immune cells in colon specimens as described in B. Exclude 15 IBD samples in C for which detailed clinical data are lacking. E) Representative SMURF2 and CD68 immunofluorescence staining of colon specimens from IBD patients and healthy controls. Scale bars, 10 µm. n = 5 per group. F) Mean fluorescence intensity (MFI) of SMURF2 in CD68^+^ macrophages as described in E, n = 5 per group. MFI was calculated according to the standards described in Section “Materials and Methods”. G) RT‐qPCR analysis of *Smurf2* mRNA expression in colon tissue from water or 2.5% DSS treated mice. H) Representative SMURF2 and F4/80 immunofluorescence staining of mice colon sections from DSS‐treated mice (Day 6), Scale bars, 10 µm. n = 4 per group. I) MFI of SMURF2 staining in F4/80^+^ macrophages as described in H. J) RT‐qPCR (left) and Western blotting analysis (right) of SMURF2 expression in immortalized bone marrow‐derived macrophages (iBMDMs) treated with fecal lysates (FLs) form control mice (CON‐FLs) or DSS‐treated mice (DSS‐FLs) for the indicated times. K,L) RT‐qPCR (left) and Western blotting analysis (right)of SMURF2 expression in iBMDMs treated with LPS (100 ng mL^−1^) (H) or peptidoglycan (40 µg mL^−1^) (l) for the indicated times. M,N) ChIP assay of histone methylation in specific regions of the SMURF2 promotor in iBMDMs treated with or without LPS or PGN for 24 h.Data are shown as mean ± SEM. Each dot represents a biological replicate (A, C, D, F, G, I) or a technical replicate (J–N). Data are representative of at least two independent experiments (D–J). P values were calculated by unpaired Student's *t*‐tests. *p < 0.05, ** *p* < 0.01, ***p < 0.001, ns, Non‐significant, *P* > 0.05. P values were calculated by 2‐tailed Student's *t* test (A, F, G, I, L, M, N) or one‐way analysis of variance (ANOVA) (C, D, J, K). See also Figure S1 (Supporting Information).

To establish the correlation of SMURF2 expression with IBD, we analyzed three RNA‐seq datasets of clinical colon samples from patients with ulcerative colitis (UC) or Crohn's disease (CD) (GSE235236, GSE53306, GSE193677). As a result, *SMURF2* was dramatically downregulated in intestinal mucosa from patients with active UC and CD compared with the normal control group (Figure S1A, Supporting Information). The down‐regulated *SMURF2* expression was only observed in active UC but not in inactive UC (Figure S1B, Supporting Information). Consistently, *SMURF2* expression was significantly reduced in inflamed mucosa compared to matched non‐inflamed mucosa (Figure S1C, Supporting Information). The anti‐inflammatory drug 5‐aminosalicylic acid (5‐ASA) is the most commonly prescribed therapy available for IBD.^[^ ^19^ ^]^ Examination of GEO data showed that patients responding to 5‐ASA treatment exhibited a higher colonic *SMURF2* gene expression than non‐responders (Figure S1D, Supporting Information). We next assessed the colonic SMURF2 expression in IBD progression via collecting clinical biopsy specimens from patients with UC or CD. RT‐qPCR analysis revealed the impaired SMURF2 expression in the inflamed mucosa versus their matched non‐inflamed counterpart (**Figure** **1**A). Immunohistochemical staining (IHC) showed that the abundance of SMURF2 was remarkably decreased within mesenchymal immune cells in both UC and CD patients versus normal individuals (Figure 1B,C). We further found much lower levels of SMURF2 in mesenchymal immune cells in patients with moderate or severe colitis than in those with mild colitis (Figure 1D).

Dysregulated macrophages and T cells are central features of IBD.^[^ ^20^, ^21^ ^]^ Therefore, we conducted immunofluorescence (IF) staining and found a significant reduction of SMURF2 expression in colonic macrophages (Figure 1E,F) but not in T cells (Figure S1E,F, Supporting Information) in IBD patients. A single‐cell RNA sequencing (scRNA‐seq) set of the colon tissue from IBD patients^[^ ^22^ ^]^ revealed the significantly impaired *SMURF2* expression in colonic macrophages from inflamed tissues when compared to that from non‐inflamed tissues (Figure S1G, Supporting Information). Further analysis of the SCP259 dataset using the online scIBD platform (http://scibd.cn/) showed that SMURF2 was enriched in APOE^+^ and LYVE1^+^ macrophages (Figure S1H, Supporting Information), which are defined as TRM.^[^ ^23^ ^]^

To evaluate whether impaired SMURF2 expression in colonic macrophages in IBD is conserved between human and mice, we challenged mice with dextran sodium sulfate (DSS) to induce colitis model and observed decreased *Smurf2* expression in the colon tissue from DSS‐treated mice (Figure 1G). Similar result was observed in a colonic tissue RNA‐seq dataset of DSS‐treated mice (Figure S1I, Supporting Information). Immunofluorescence staining further revealed that the protein levels of SMURF2 were reduced in colonic macrophages from DSS‐treated mice compared to normal control mice (Figure 1H,I). In line with enriched SMURF2 expression in human colon TRMs (Figure S1H, Supporting Information), flow cytometry analysis revealed higher expression of SMURF2 in TRMs (CD11b^+^CX3CR1^hi^) than that in neutrophils (CD11b^+^Ly6G^+^), monocytes (CD11b^+^ Ly6C^+^) and MDMs (CD11b^+^CX3CR1^int^) (Figure S1J,K, Supporting Information). Cx3cr1^CreERT2/+^: R26^tdTomato^ reporter mice were treated with DSS and we observed DSS administration markedly reduced SMURF2 expression in colonic TRMs (Figure S1L,M, Supporting Information). Taken together, these data suggest that the reduced SMURF2 expression in colonic macrophages is associated with IBD.

We next sought to elucidate the mechanism underlying the impaired SMURF2 expression in TRMs during colitis. It has been reported that disruptions to the intestinal barrier in colitis facilitate the invasion of bacteria and their components or metabolites into the colon lamina propria (CLP), leading to the activation of local immune cells, such as macrophages.^[^ ^24^ ^]^ As shown in Figure S1N–P (Supporting Information), a broad‐spectrum antibiotic combination (ABX) treatment abolished the reduction of SMURF2 expression in colonic TRMs caused by DSS treatment, indicating gut microbiota are responsible for the downregulation of SMURF2 expression in colitis mice. We then prepared fecal samples pooled from control or DSS‐treated mice and performed fecal microbiota transplantation (FMT) in microbiota‐depleted ABX mice (Figure S1Q, Supporting Information). Neither DSS nor control feces affected the SMURF2 expression in the colonic TRMs (Figure S1R,S, Supporting Information). However, both control fecal lysates (Con‐Fls) and DSS‐treated mice fecal lysates (DSS‐Fls) treatment led to a comparable reduction of *Smurf2* RNA and protein levels in the immortalized bone marrow‐derived macrophages (iBMDMs) (Figure 1J). Collectively, these findings indicate that increased intestinal mucosal permeability leads to gut microbial invasion, resulting in the reduction of SMURF2 expression in colonic macrophages during colitis.

Gut microbiota regulates colonic macrophages via PAMPs and metabolites such as short‐chain fatty acids (SCFA) and lactate.^[^ ^25^ ^]^ We then treated macrophages with these compounds individually and observed that treatment with both Gram‐negative bacteria‐derived LPS and Gram‐positive bacteria‐derived PGN suppressed SMURF2 expression (Figure 1K,L). However, neither SCFA nor lactate treatment affects SMURF2 expression (Figure S1T,U, Supporting Information). Histone modifications are associated with the transcriptional regulation of genes, among which K4 and K36 are methylated in transcriptionally active chromatin, whereas methylation of K9 and K27 is linked with inactive chromatin.^[^ ^26^ ^]^ By chromatin immunoprecipitation (ChIP) assay, we found that H3K4me3 and H3K36me3 were downregulated in the promoter region of SMURF2 in iBMDMs after LPS or PGN stimulation, whereas H3K9me3 and H3K27me3 remained constant throughout the stimulation (Figure 1M,N). In addition, the treatment of H3K4me3 inhibitor OICR‐9429^[^ ^27^ ^]^ or H3K36me3 inhibitor EZM0414^[^ ^28^ ^]^ led to a significant reduction in SMURF2 expression (Figure S1V, Supporting Information). Taken together, these findings indicate that the invaded microbes release PAMPs to diminish the expression of SMURF2 in TRMs and may also involve epigenetic modifications of histone H3.

### 2.2 Myeloid‐Specific *Smurf2* Deficiency Aggravates Experimental Colitis

**Figure 2:**
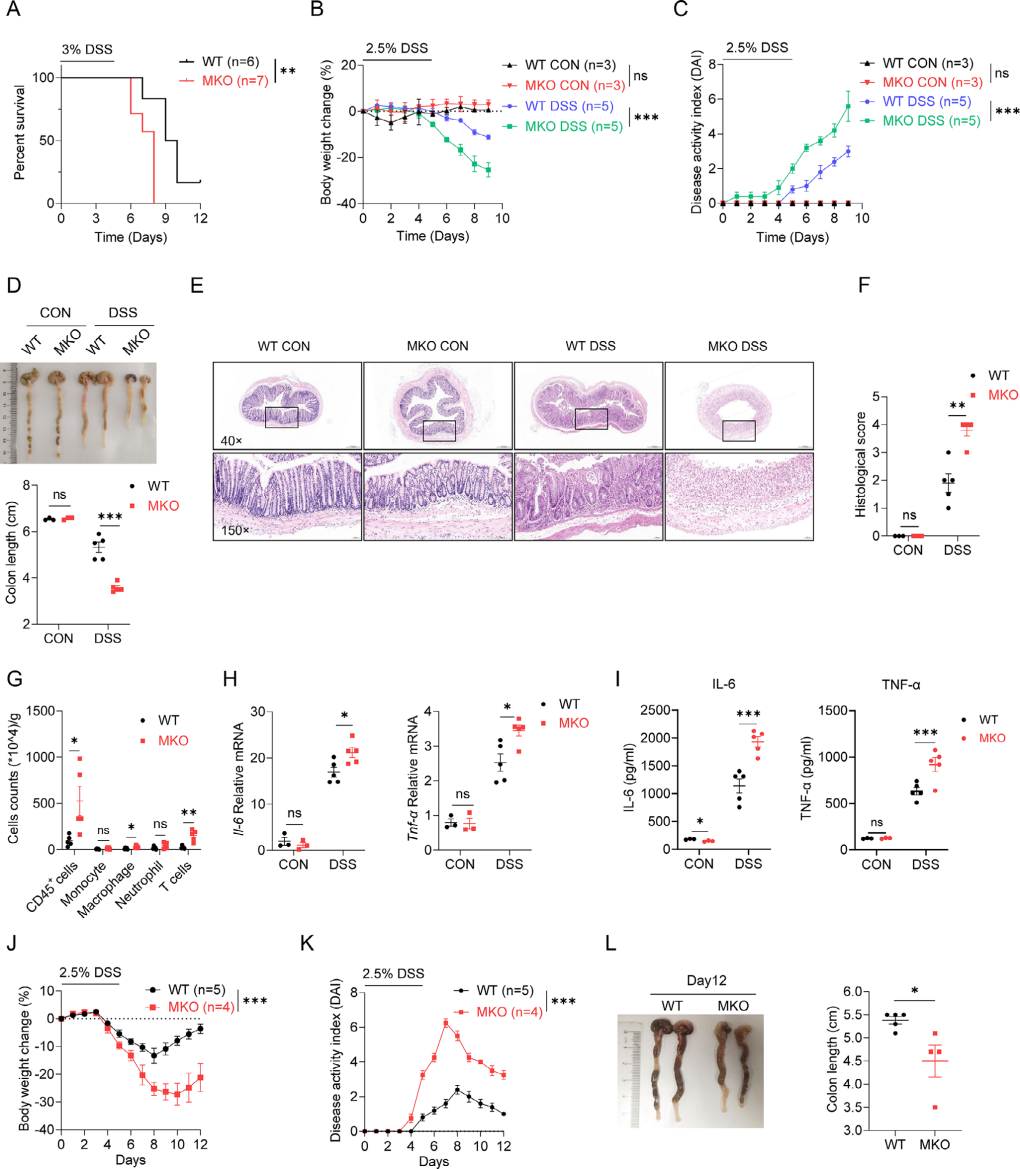
*Smurf2* deficiency in myeloid cells aggravates experimental colitis. A) *Smurf2* myeloid knockout (MKO) mice and control wide type (WT) mice were administered 3% DSS for 5 days followed by water feeding to induce severe acute colitis. Mouse death was monitored until day 12. B–I) WT and MKO mice were administered 2.5% DSS for 5 days followed by 4 days water to induce acute colitis. Mice were sacrificed on day 9. Body weight change (B) and disease activity index (DAI) (C) were assessed daily. Gross morphology images of the colon (up) and colon length (down) were measured on day 9 (D), representative H&E staining (E) and histopathological score of colonic sections (F) were assessed as indicated protocol. Flow cytometry analysis of cell numbers of the colon lamina propria (CLP)‐infiltrated immune cells (G), RT‐qPCR (H) and ELISA analysis (I) of colonic IL‐6 and TNF‐α expression. J–L) WT and MKO mice were treated with 2.5% DSS for 5 days and then fed with water for 7 days. Body weight change(J) and DAI (K) were assessed daily. Gross morphology images of the colon (left) and colon length (right) were measured on day 12 (L). Data are shown as mean ± SEM. Each dot represents a biological replicate (D, F, G, H, I, L), the n values also represent the number of biological replicates (A, B, C, J, K). All experiments were performed at least two times. *p < 0.05, ** *p* < 0.01, ***p < 0.001, ns, Non‐significant, *P* > 0.05. P values were calculated by using Log‐rank (Mantel–Cox) test (A), or two‐way ANOVA (B, C, J, K) or 2‐tailed Student's *t* test (D, F, G, H, I). See also Figure S2 (Supporting Information).

To further assess the role of macrophage‐derived SMURF2 in colitis, myeloid cell conditional knockout mice (referred to as MKO) were generated by crossing *Smurf2^f/f^* (referred to as WT) mice with *Lyz2‐iCre* mice. The MKO mice and their WT littermates exhibited comparable frequencies of monocytes, macrophages and neutrophils in spleens and CLP (Figure S2A, B, Supporting Information). Subsequently, an experimental colitis mouse model was established by treating mice with DSS. MKO mice showed a significantly decreased survival rate compared with WT littermates after 3% DSS treatment (**Figure** **2**A). To monitor disease progression, MKO and WT mice were administered 2.5% DSS for 5 days, followed by regular water. When compared to their WT littermates, MKO mice displayed greater body weight loss (Figure 2B), higher disease activity index (DAI) (Figure 2C), and shorter colon length (Figure 2D). In line with these findings, hematoxylin and eosin (H&E) staining showed a dramatically exacerbated colonic histopathology in MKO mice, characterized by inflammatory cell infiltration, epithelial damage, and ulceration formation (Figure 2E,F). Flow cytometry analysis revealed enhanced accumulation of CD45^+^ immune cells including CD11b^+^F4/80^+^ macrophages, CD11b^+^Ly6C^+^ monocytes, and T cells in the colonic tissue from MKO versus their WT littermates (Figure 2G; Figure S2C,D, Supporting Information). Meanwhile, *Smurf2* deficiency led to a significantly increased mRNA expression (Figure 2H) and secretion of pro‐inflammatory cytokines IL‐6 and TNF‐α (Figure 2I).

The DSS‐induced IBD consists of two stages: acute (day 5) stage and recovery (days 9–20) stage.^[^ ^29^ ^]^ During the recovery period of intestinal inflammation, MKO mice exhibited a slower rate of body weight gain and a higher DAI than WT littermates (Figure 2J,K). Moreover, MKO mice had shorter colons than their WT littermates on day 12 (Figure 2L). H&E staining revealed more severe colonic epithelial damage and higher histological score in MKO mice than WT mice (Figure S2E,F, Supporting Information). These findings suggest the protective role of myeloid *Smurf2* in DSS‐induced experimental colitis.

Dysbiosis of gut microbiota is associated with intestinal diseases such as IBD and irritable bowel syndrome.^[^ ^30^ ^]^ To determine whether the increased susceptibility to experimental colitis in MKO mice is associated with gut microbiota, we collected feces from *Smurf2* WT and MKO mice and conducted 16S rRNA sequencing. As shown in Figure S2G,H (Supporting Information), no significant differences were observed in intestinal microbiota diversity between the *Smurf2* WT and MKO groups, indicating that *Smurf2* knockout does not affect intestinal microbiota. WT and MKO mice were cohoused to establish fecal microbiota transfer between the mice. During the period of colitis induction, cohoused MKO mice still showed exacerbated colitis symptoms (Figure S2I,J, Supporting Information) and shorter colon length (Figure S2K, Supporting Information) compared to cohoused WT mice. Consistently, MKO mice exhibited more severe colitis symptoms after depletion of the intestinal microbiota with ABX (Figure S2L‐N, Supporting Information). These results indicate that the increased susceptibility to DSS‐induced colitis in MKO mice was microbiota‐independent.

### 2.3 *Smurf2* Deficiency Promotes TRM Proliferation in the Early Stage of Colon Inflammation

**Figure 3:**
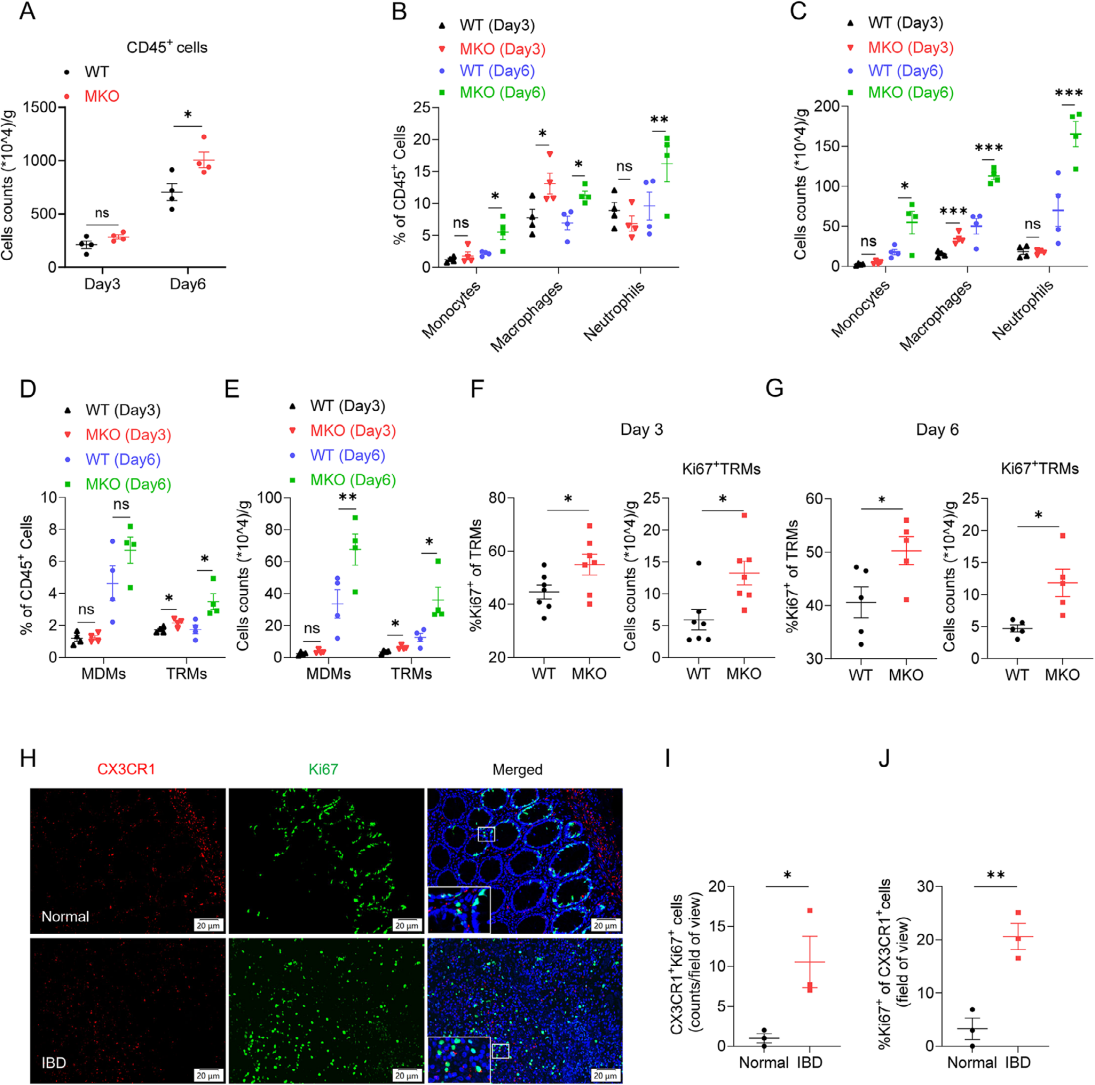
*Smurf2* deficiency promotes TRM proliferation. A–E) *Smurf2* WT and MKO mice were fed with 2.5% DSS and then sacrificed on day 3 or 6 (DSS fed for 5 days), followed by flow cytometry analysis of colon‐infiltrated immune cells, n = 4 per group. Cell numbers of CD45^+^ immune cells (A). Quantified percentages (B) and cell numbers (C) of myeloid cells. Quantified percentages (D) and cell numbers (E) of MDMs and TRMs myeloid cells. F,G) Flow cytometry analysis of percentages and cell number of Ki67^+^ in colonic TRM from WT and MKO mice on day 3 after colitis induction (n = 7 per group) (F) or day 6 (n = 5 per group) (G) of colitis induction. H–J) Representative CX3CR1 and Ki67 immunofluorescence staining of colon biopsy from normal control and IBD patients (H). Scale bars, 20 µm. n = 3 per group. Quantitative analysis of CX3CR1 and Ki67 double positive cells (I) and percentage of Ki67^+^ cells in CX3CR1^+^ cells (J). Data are shown as mean ± SEM. Each dot represents a biological replicate (A‐G, I, J). Data are representative of at least two independent experiments. *p < 0.05, ** *p* < 0.01, ns, Non‐significant, *P* > 0.05. P values were calculated by using 2‐tailed Student's *t* test (A, F, G, I, J), or one‐way ANOVA (B, C, D, E), See also Figure S3 (Supporting Information).

To investigate the underlying mechanism of myeloid SMURF2 in protecting mice from experimental colitis, we conducted a time‐course analysis of myeloid cell subpopulations in CLP upon DSS treatment. Epithelial damage and colon length shortening appeared on the fourth day following DSS treatment.^[^ ^31^ ^]^ Therefore, we challenged MKO mice and their WT littermates with DSS and sacrificed the mice on day 3 or 6. Consistently, both WT and MKO mice exhibited normal colon appearances, with no signs of bloody or loose stools on day 3 (Figure S3A, Supporting Information). H&E staining showed there was no intestinal epithelial damage in either WT or MKO mice on day 3 (Figure S3B, Supporting Information). Whereas, the mice showed obvious colon shortening and intestinal bleeding 6 days after DSS treatment. More importantly, MKO mice exhibited shorter colon (Figure S3A, Supporting Information) and higher histological score (Figure S3B, Supporting Information), as well as enhanced accumulation of immune cells (**Figure** **3**A) when compared to WT littermates. Although MKO mice showed a comparable accumulation of CD45^+^ immune cells in the lamina propria with WT mice on day 3 of DSS treatment (Figure 3A), we observed elevated accumulation of macrophages in MKO versus WT colon (Figure 3B,C; Figure S3C, Supporting Information). And on day 6, the frequency of monocytes, macrophages and neutrophils were significantly increased in MKO mice (Figure 3B,C; Figure S3D, Supporting Information). Interestingly, Myeloid‐specific *Smurf2* deficiency significantly promoted the accumulation of TRMs without affecting the infiltration of MDMs on day 3 (Figure 3D,E). Both TRM and MDM accumulation were enhanced in MKO mice versus WT mice on day 6 (Figure 3E). More importantly, MKO mice showed a higher proportion and cell number of Ki67^+^ TRMs than WT mice (Figure 3F,G), but a comparable frequency of DAPI^+^ TRMs (Figure S3E, Supporting Information). Notably, *Smurf2* deficiency had no effect on the accumulation of TRMs in naive mice (Figure S3F, Supporting Information). Immunofluorescence staining also showed the enhanced proliferation of colonic TRMs in patients with IBD (Figure 3H–J). Macrophages derived TNF‐α has been recognized as the main inflammatory cytokines at the early stage of DSS‐induced colitis.^[^ ^32^ ^]^ As shown in Figure S3G (Supporting Information), there was no difference in the expression of TNF‐α in TRMs between WT and MKO mice. M‐CSF is the major cytokine responsible for the proliferation of intestinal TRMs.^[^ ^33^ ^]^ RT‐qPCR and ELISA analysis revealed a comparable expression of M‐CSF(*Csf1*) between WT and MKO colon tissue (Figure S3H,I, Supporting Information). Meanwhile, *Smurf2* deficiency did not affect CSF1R expression in BMDMs, colonic MDMs, or TRMs from day 3 and day 6 colitis mice (Figure S3J–M, Supporting Information). Taken together, these results suggest that *Smurf2* deficiency promotes TRMs proliferation and accelerates the progression of experimental colitis.

### 2.4 Colonic TRM‐Derived SMURF2 Alleviates DSS‐Induced Experimental Colitis

**Figure 4:**
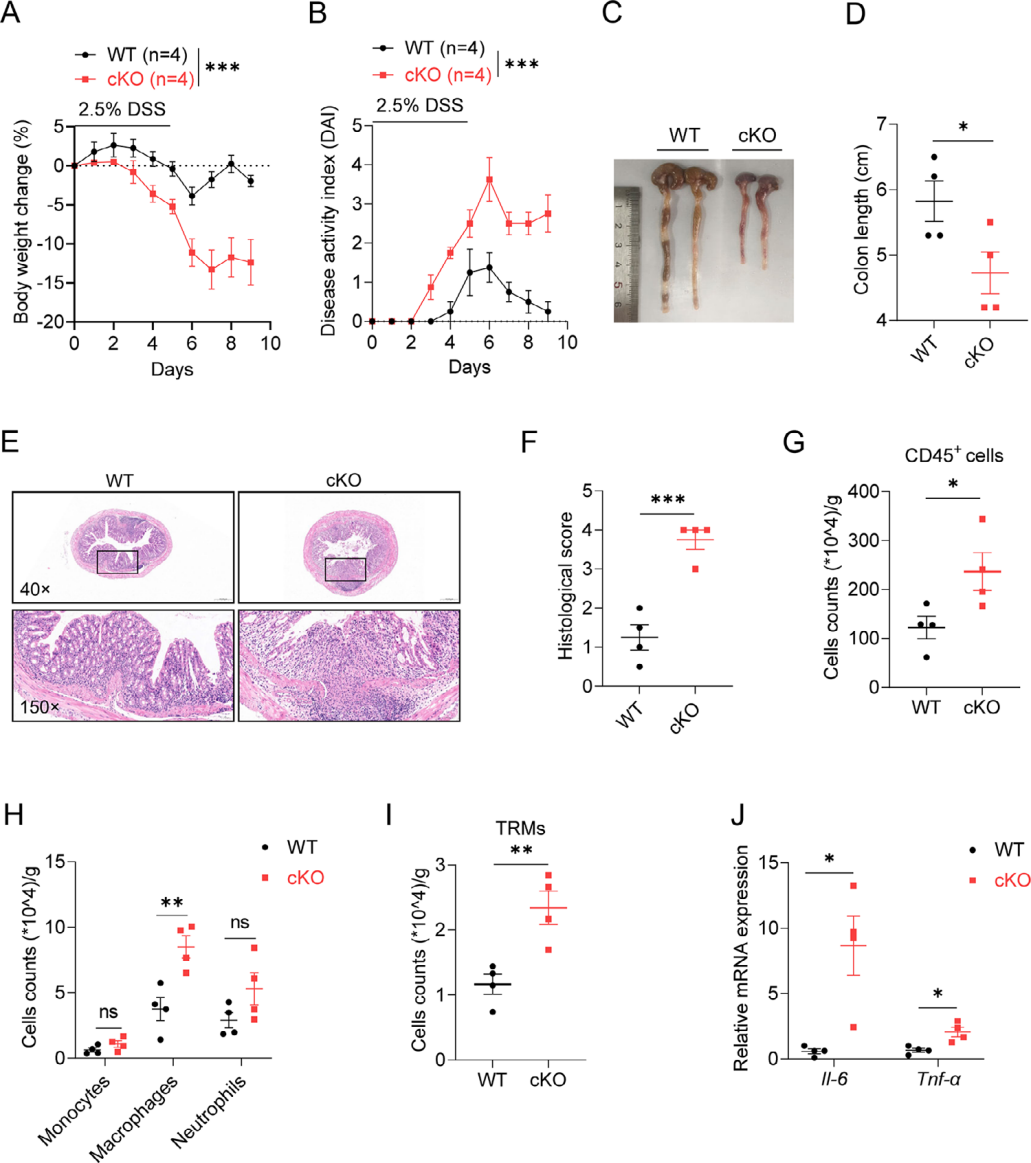
TRM‐specific *Smurf2* deficiency exacerbates experimental colitis. A–D) WT and cKO mice were administered 2.5% DSS for 5 days followed by 4 days water, and mice were sacrificed on day 9. Body weight change(A) and disease activity index (DAI) (B) were assessed daily. Gross morphology images (C) and colon length were measured on day 9 (D). E,F) Representative H&E staining (E) and histopathological score (F) of colon tissue from WT or cKO mice as described in A. G–I) Flow cytometry analysis of the cell numbers of CD45^+^ immune cells (G), myeloid cells (H), TRMs (I) in colonic lamina propria from WT or cKO mice as described in A. J) WT and cKO mice were administered 2.5% DSS for 5 days followed by 4 days water, and mice were sacrificed on day 9. RT‐qPCR analysis of *Il‐6* and *Tnf‐α* expression in colon tissue from DSS‐treated WT and cKO mice. Data are shown as mean ± SEM. Each dot represents a biological replicate (D,F–J), the n values also represent the number of biological replicates (A,B). Data are representative of at least two independent experiments. *p < 0.05, ** *p* < 0.01, ***p < 0.001, ns, Non‐significant, *P* > 0.05. P values were calculated by using two‐way ANOVA (A, B) or 2‐tailed Student's *t* test (D, F–J). See also Figure S4 (Supporting Information).

To further confirm the role of TRM cell‐intrinsic SMURF2 in intestinal homeostasis, we crossed *Smurf2^f/f^* mice with *Cx3cr1^Cre‐ERT^* mice to enable specific gene knockout in TRMs (referred to as cKO).^[^ ^14^ ^]^ Six weeks after tamoxifen treatment, flow cytometry confirmed the specific deletion of *Smurf2* in colon TRMs, but not in monocytes in cKO mice (Figure S4A,B, Supporting Information). Of note, there is no difference in frequency and number of colonic TRMs between cKO and WT littermates (Figure S4C,D, Supporting Information). Consistently, compared to their WT counterparts, cKO mice exhibited significantly greater body weight loss (**Figure** **4**A), higher DAI (Figure 4B), shorter colon length (Figure 4C,D), and enhanced epithelium disruption and immune cell infiltration after DSS treatment (Figure 4E,F). Flow cytometry analysis revealed an increased accumulation of CD45^+^ immune cells (Figure 4G), macrophages (Figure 4H), and TRMs (Figure 4I) in the colonic lamina propria from cKO versus WT mice. Q‐PCR analysis identified more enrichment of pro‐inflammatory cytokine *Il‐6* and *Tnf‐α* in the cKO colon tissue (Figure 4J, Supporting Information). These results indicate that TRM‐derived SMURF2 alleviates experimental colitis.

### 2.5 Microglia‐Derived *Smurf2* Alleviates CNS Autoimmune Inflammation

**Figure 5:**
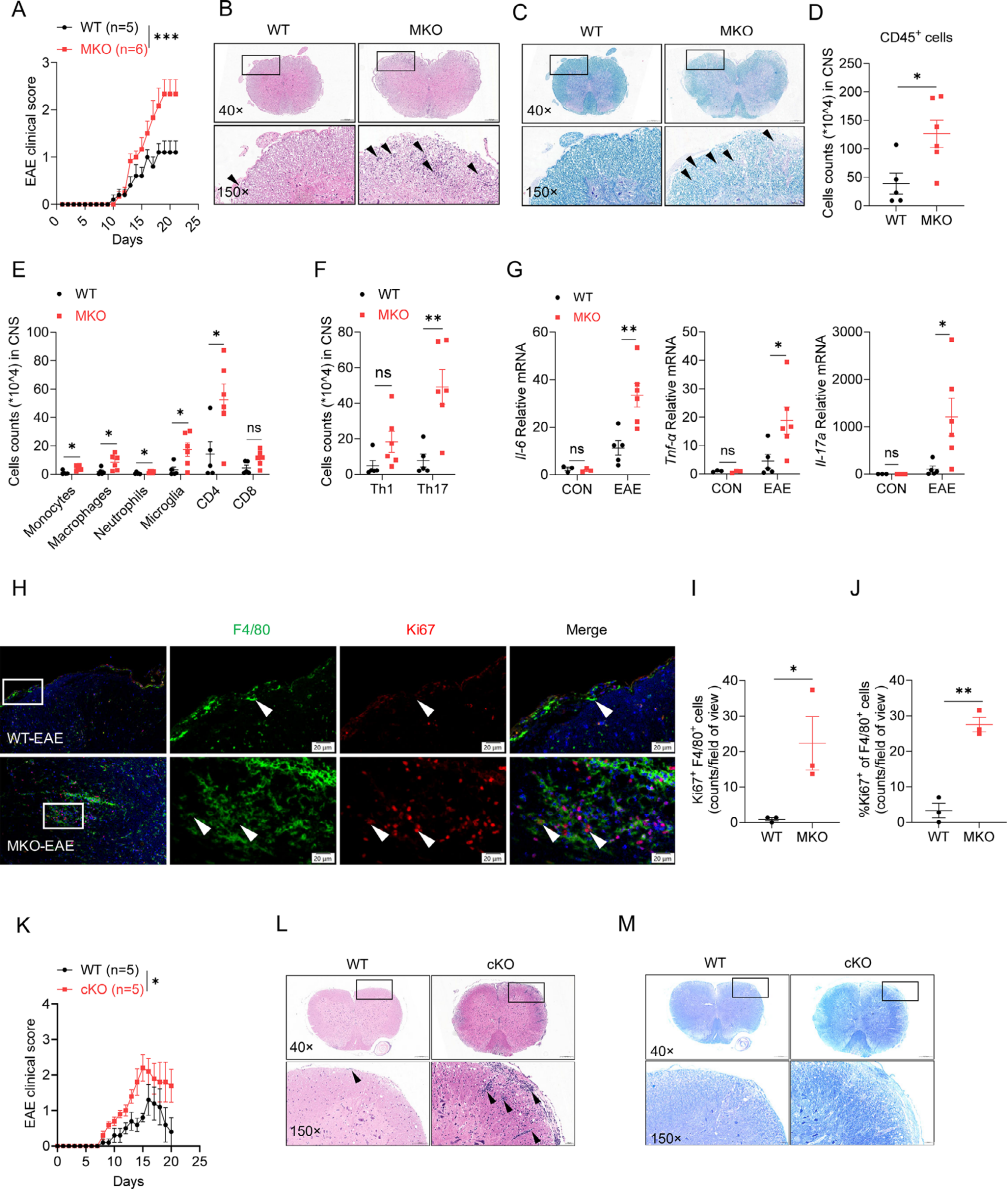
Microglia‐specific *Smurf2* deficiency promotes central nervous system (CNS) autoimmune inflammation. A) WT and MKO mice were immunized with MOG_35‐55_ for 20 days to induce EAE. Mean clinical scores were calculated every day according to the standards as described in Section “Materials and Methods”. B,C) Representative H&E staining (B) and Luxol fast blue (LFB) staining (C) of spinal cord sections from WT (n = 5) and MKO (n = 5) as described in A. D–F) Flow cytometry analysis of cell numbers of CD45^+^ immune cells (A), myeloid cells and T cells (B) and Th1 and TH17 cell (F) in CNS from WT and MKO mice described in A. G) RT‐qPCR analysis of *Il‐6*, *Tnf‐α* and *Il‐17a* expression in CNS tissue from WT and MKO mice as described in A. H–J) Representative Ki67 and F4/80 immunofluorescence staining (H), number of Ki67^+^F4/80^+^ cells (I) and percentage of Ki67^+^ in F4/80^+^ macrophages J) in the mice spinal cord sections from WT and MKO EAE mice. Scale bars, 20 µm. n = 3 per group. K–M) WT and cKO mice were immunized with MOG_35‐55_ to induce EAE using the same method as in A. Mean clinical scores were calculated every day (K). Representative H&E staining (L) and LFB staining (M) of the spinal cord from WT and cKO EAE mice. n = 5 per group. Data are shown as mean ± SEM. Each dot represents a biological replicate (D‐G, I, J), the n values also represent the number of biological replicates (A, K). Data are representative of at least two independent experiments. *p < 0.05, ** *p* < 0.01, ***p < 0.001, ns, Non‐significant, *P* > 0.05. P values were calculated by using two‐way ANOVA (A, K) or 2‐tailed Student's *t* test (D‐G, I, J). See also Figure S5 (Supporting Information).

As TRMs in the CNS, microglia are critical in the initiation and progression of neurodegenerative diseases and multiple sclerosis.^[^ ^34^ ^]^ We observed the enrichment of *SMURF2* in microglia and astrocytes by analyzing single‐cell RNA‐sequencing data in the Human Protein Atlas (Figure S5A, Supporting Information). Examination of the GEO database (GSE131282) unveiled a significant downregulation of *SMURF2* gene expression in grey matter from patients with PPMS (primary progressive MS) and SPMS (secondary progressive MS) (Figure S5B, Supporting Information). MKO mice displayed no signs of spontaneous inflammation or demyelinating changes (Figure S5C,D, Supporting Information). Consistently, no significant differences in frequency and number of microglia were observed between cKO and their WT littermates (Figure S5E,F, Supporting Information). We then immunized MKO mice and their littermates with a myelin oligodendrocyte glycoprotein (MOG) peptide (MOG_35–55_) along with pertussis toxin (PTX) to induce CNS autoimmune model EAE. As shown in **Figure** **5**A, MKO mice suffered from relatively severe EAE disease symptoms and had higher EAE clinical scores when compared to their WT counterparts. Moreover, MKO mice displayed increased inflammatory cell infiltration (Figure 5B) and demyelination (Figure 5C), as demonstrated by hematoxylin‐eosin and luxol fast blue (LFB) staining, respectively. Consistently, flow cytometry analysis of mouse CNS tissues (brains and spinal cords) showed that infiltration of CD45^+^ immune cells, especially myeloid cells (monocytes, macrophages and neutrophils), microglia (CD11b^+^CD45^low^), and CD4^+^ T were significantly increased in MKO mice compared to WT mice (Figure 5D,E; Figure S5H, Supporting Information). Within the CNS infiltrating CD4^+^ T‐cell population, the accumulation of Th17 (IL‐17^+^) but not Th1 cells (IFNγ^+^) was significantly increased in MKO mice (Figure 5F; Figure S5G, Supporting Information). Additionally, MKO mice showed higher expression of proinflammatory cytokines, including *Il‐6*, *Tnf‐α*, and *Il‐17a* within the CNS than WT mice (Figure 5G). More importantly, Ki67 immunofluorescence staining revealed an elevated presence of Ki67^+^ macrophages within the CNS in MKO versus WT mice (Figure 5H–J), indicating that *Smurf2* deficiency increases macrophage proliferation during the progression of EAE.

To further determine whether *Smurf2* deficiency in microglia contributes to EAE pathogenesis. We induced CNS autoimmunity model EAE in TRM‐conditional *Smurf2*‐knockout mice (cKO), which specifically deleted *Smurf2* in microglia without affecting its expression in BMDMs (Figure S5I, Supporting Information). Parallel to MKO mice, cKO mice displayed accelerated disease progression and enhanced pathological changes compared to their WT littermates (Figure 5K,M), as well as elevated immune cell infiltration (Figure S5J, Supporting Information) and expression of pro‐inflammatory cytokines, including *Il‐6* and *Tnf‐α* in the CNS (Figure S5K, Supporting Information). Collectively, these results indicate that microglia‐specific *Smurf2* deficiency aggravates CNS autoimmune inflammation.

### 2.6 *Smurf2* Deficiency Promotes Macrophage Proliferation and Aggravates Autoimmune Inflammation via Downregulating the Levels of p‐TBK1

**Figure 6:**
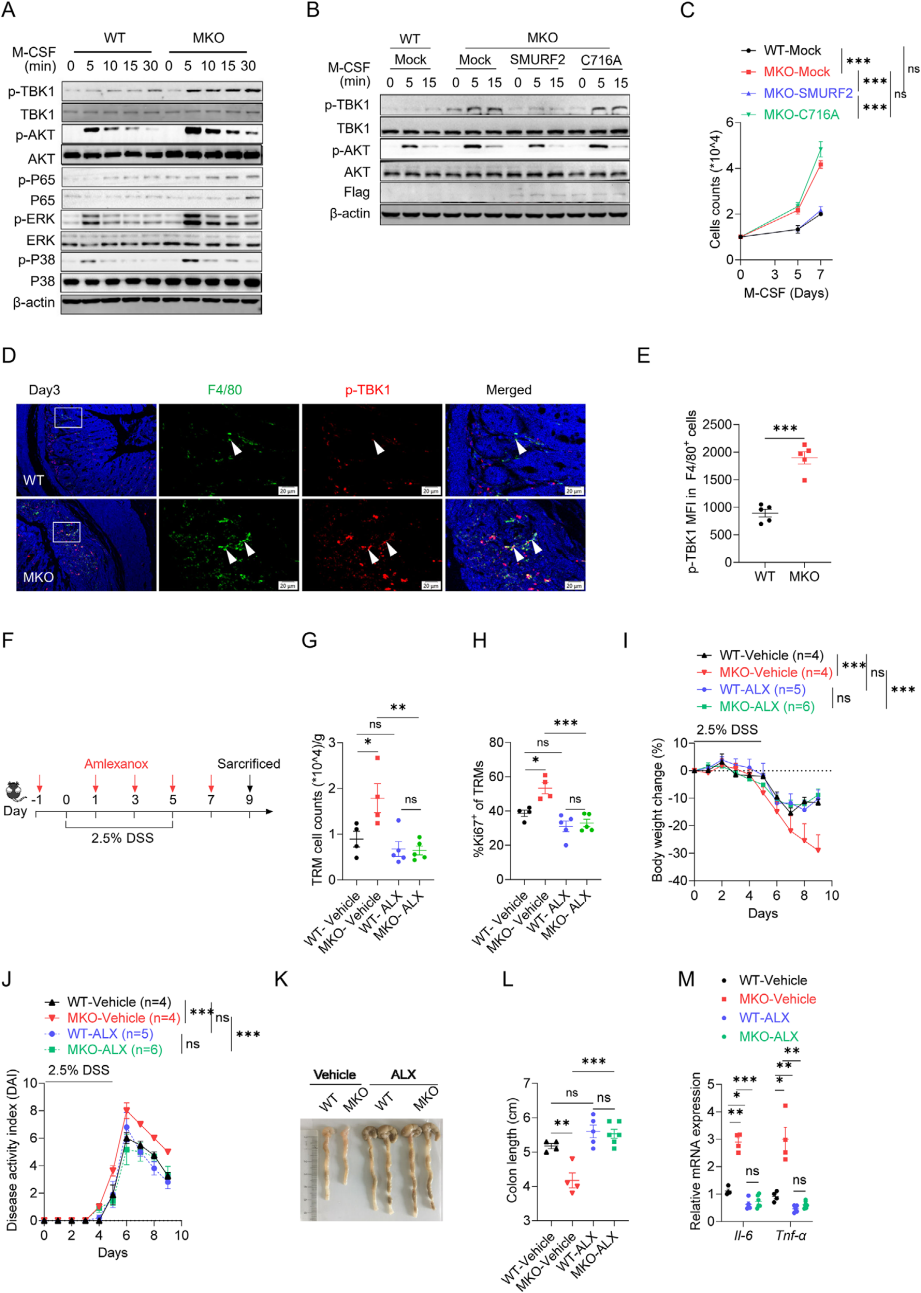
*Smurf2* deficiency promotes macrophage proliferation and aggravates autoimmune inflammation in a TBK1‐dependent manner. A) Western blotting analysis of TBK1/AKT/NF‐kB/MAPK activation in WT and MKO BMDMs treated with M‐CSF (100 ng mL^−1^) for the indicated times. B) Western blotting analysis of TBK1/AKT activation in WT and SMURF2 or C716A mutant reconstructed MKO BMDMs treated with M‐CSF for the indicated times. C) Cell proliferation analysis of WT, MKO and MKO bone marrow cells transduced with SMURF2 or its C716A mutant cultured in M‐CSF (20 ng mL^−1^) for the indicated times. D) Representative p‐TBK1 and F4/80 immunofluorescence of colon sections from DSS‐treated mice (Day 3). Scale bars, 20 µm. n = 5 per group. E) MFI of p‐TBK1 in F4/80^+^ macrophages as described in D. F) Schematic diagram of ALX treatment. WT and MKO mice were orally administrated TBK1 inhibitor ALX at a dose of 25 mg kg^−1^, along with 2.5% DSS in drinking water. Mice were sacrificed on day 3 or day 9. G,H) Flow cytometry analysis of cell numbers of TRMs and quantified percentages of Ki67^+^ TRM (G) in CLP on day 3 of colitis induction, with or without ALX administration. I–M) WT and MKO mice were treated as described in F and scarified on day 9. The body weight changes (I), DAI (J), gross morphology images (K) and colon length (L). The expression of *Il‐6* and *Tnf‐α* was assessed by RT‐qPCR (M) Data are shown as mean ± SEM. Each dot represents a biological replicate, the n values also represent the number of biological replicates. Data are representative of at least two independent experiments. *p < 0.05, ** *p* < 0.01, ***p < 0.001, ns, Non‐significant, *P* > 0.05. P values were calculated by using two‐way ANOVA (C, I, J) or 2‐tailed Student's *t* test (E) or one‐way ANOVA (G, H, L, M). See also Figure S6 (Supporting Information).

To address the potential role of SMURF2 in macrophage proliferation, we cultured bone marrow cells (BMCs) from WT and MKO mice with M‐CSF. Results showed that MKO macrophages exhibited enhanced colony formation (Figure S6A,B, Supporting Information) and accelerated proliferation (Figure S6C,D, Supporting Information) compared with WT macrophages. Moreover, *Smurf2* deficiency promotes the expression of proliferation‐related genes, specifically *c‐Myc* and *CyclinD1* (Figure S6E, Supporting Information). To dissect the mechanistic role of SMURF2 in macrophage proliferation, comparative RNA‐Seq analysis was performed between M‐CSF‐treated WT and MKO BMDMs in‐house. Based on the Gene Ontology (GO) enrichment analysis, MKO BMDMs showed an enhanced innate immune response signaling when compared to WT BMDMs, in which TBK1‐is the key molecule^[^ ^35^ ^]^ (Figure S6F, Supporting Information). The Search Tool for the Retrieval of Interacting Genes/Proteins (STRING) predicted the interaction between SMURF2 and TBK1 (Figure S6G, Supporting Information). Indeed, *Smurf2* deficiency significantly enhanced the phosphorylation of TBK1 in M‐CSF‐treated BMDMs (**Figure** **6**A). M‐CSF treatment activates AKT and downstream NF‐κB/MAPK signaling to induce macrophage proliferation and survival.^[^ ^36^ ^]^ We observed enhanced activation of these signals in MKO macrophages (Figure 6A). SMURF2 but not SMURF2‐C716A mutant, which lacks ubiquitin ligase activity,^[^ ^37^ ^]^ abolished the increased M‐CSF‐induced TBK1/AKT phosphorylation (Figure 6B) and proliferation (Figure 6C) resulting from *Smurf2* knockout, indicating that the inhibitory effects of SMURF2 on M‐CSF signaling depend on its E3 ubiquitin ligase activity. A previous study reported TBK1 directly phosphorylated AKT1 and triggered the proliferation signal.^[^ ^38^ ^]^ Consistently, treatment with Amlexanox (ALX), a specific TBK1 inhibitor,^[^ ^39^ ^]^ abrogated the increased colony formation and proliferation (Figure S6H,I, Supporting Information), elevated levels of *c‐Myc* and *CyclinD1* (Figure S6J, Supporting Information), and enhanced AKT activation (Figure S6K, Supporting Information) caused by *Smurf2* deficiency. Similarly, *Tbk1* knockdown also mitigated the enhanced proliferation of iBMDMs caused by *Smurf2* deficiency (Figure S6L,M, Supporting Information).

We then conducted immunofluorescence staining on colonic tissues from DSS‐treated mice and observed elevated levels of p‐TBK1 in MKO colonic macrophages compared to WT colonic macrophages (Figure 6D,E). ALX was subsequently used to validate whether uncontrolled TBK1 signaling in MKO colonic macrophages accounted for the exacerbated colitis (Figure 6F). Administration of ALX abolished the difference in the accumulation and proliferation (Figure 6G,H) of MKO and WT TRMs. Moreover, the aggravated colitis phenotype caused by *Smurf2* deficiency was diminished after ALX treatment (Figure 6I–L; Figure S6N,O, Supporting Information), as well as the expression of pro‐inflammatory cytokines (Figure 6M).

Similarly, enhanced levels of p‐TBK1 were observed in the MKO macrophage within the CNS from EAE mice (Figure S6P,Q, Supporting Information). Consistent with previous reports,^[^ ^40^ ^]^ ALX treatment resulted in reduced clinical symptoms and pro‐inflammatory cytokines expression, but also substantially abolished the difference in EAE progression between MKO and WT mice (Figure S6R–V, Supporting Information). Consistently, ALX treatment abolished the difference in the severity of colitis (Figure S6W–AA, Supporting Information) and EAE progression (Figure S6AB–AC, Supporting Information) between cKO and WT mice. Taken together, these findings indicate that *Smurf2* ablation promotes the proliferation of TRM and accelerates autoimmune inflammation via TBK1 signaling.

### 2.7 SMURF2 Interacts with p‐TBK1 and Mediates Ubiquitination of p‐TBK1

**Figure 7:**
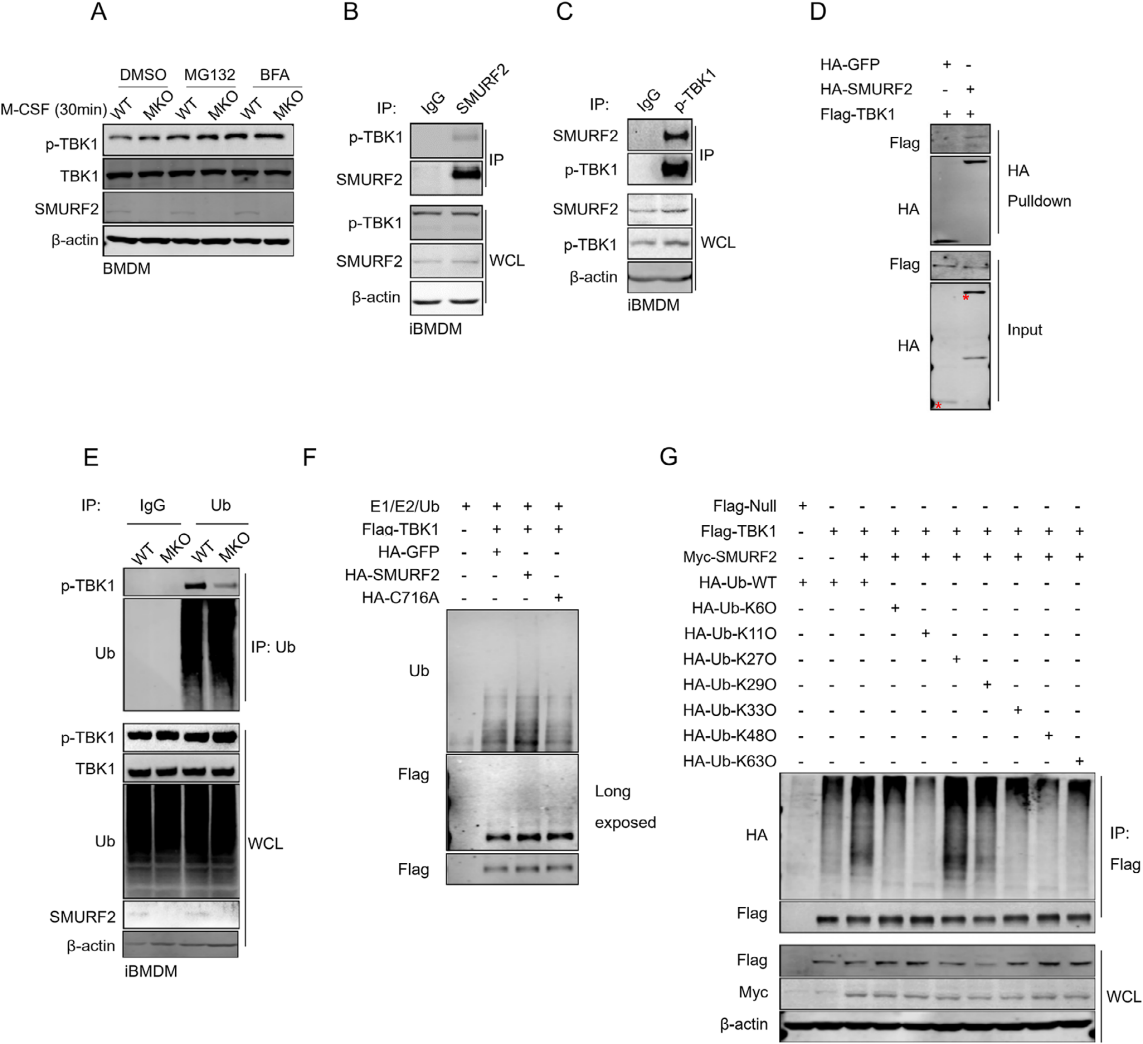
SMURF2 mediates Lys‐27‐linked polyubiquitination of p‐TBK1. A) Western blot analysis of p‐TBK1 level in M‐CSF‐stimulated WT and MKO BMDM cells which were pretreated with MG132 (10 µm) or BFA (0.2 µm) for 6 h. (B, C) Anti‐SMURF2 B) and Anti‐p‐TBK1 C) Co‐IP of the interaction between SMURF2 and p‐TBK1 in iBMDMs treated with BFA for 6 h. D) In vitro pull‐down assay of the interaction between TBK1 and SMURF2. E) Western blot analysis of ubiquitination of p‐TBK1 in WT and MKO iBMDMs treated with BFA for 6 h. F) In vitro ubiquitination analysis of TBK1. G) Ubiquitination assay of Flag‐TBK1 following immunoprecipitating TBK1 with anti‐Flag antibody from lysates of HEK293T co‐transfected with plasmids expressing Flag‐TBK1, Myc‐SMURF2, and HA‐Ub, or its mutants expressing only one lysine (K only, KO). All experiments were performed at least two times. See also Figure S7 (Supporting Information).

NEDD4 family E3 ligases have been reported to catalyze the ubiquitination of phosphorylated proteins and mediate their degradation.^[^ ^41^ ^]^ SMURF2 knockout upregulated the level of p‐TBK1 in macrophages, without affecting the total TBK1 levels (Figure 6A). We therefore investigated whether SMURF2 regulated the degradation of the p‐TBK1. WT and MKO BMDMs were treated with proteasome inhibitor (MG132) or autophagy inhibitor (Brefeldin A, BFA). Western blot showed that only BFA treatment abrogated the increased level of p‐TBK1 in MKO BMDMs (**Figure** **7**A). Consistently, overexpression of SMURF2 in RAW264.7 led to an impaired p‐TBK1 level, and this effect could be blocked by BFA but not by MG132 (Figure S7A, Supporting Information). These results indicate that SMURF2 regulates p‐TBK1 expression through the lysosomal pathway. Co‐immunoprecipitation (Co‐IP) assay showed the interaction between endogenous SMURF2 and p‐TBK1 in iBMDMs (Figure 7B,C). It has been reported that the TBK1 S172A mutation abrogates auto‐phosphorylation of TBK1.^[^ ^42^ ^]^ In the transiently overexpressed HEK293T cells, the CO‐IP result indicated that SMURF2 coprecipitated with TBK1 but not the S172A mutation (Figure S7B,C, Supporting Information), indicating SMURF2 specifically interacts with p‐TBK1. To map the domains required for SMURF2 to interact with TBK1, we constructed a series of plasmids expressing wild‐type or truncation mutants, in which the C2 (SMURF2‐ΔC2, dC2), WW (SMURF2‐ΔWW, dWW), or HECT (SMURF2‐ΔHECT, dHECT) domains were deleted.^[^ ^43^ ^]^ As shown in Figure S7D (Supporting Information), deletion of the WW or HECT domain, but not the C2 domain, disrupted the interaction between SMURF2 and TBK1. In vitro pull‐down assay confirmed that SMURF2 directly bound with TBK1 (Figure 7D).

As SMURF2 is a HECT E3 ubiquitin ligase, we then examined whether SMURF2 catalyzes the ubiquitination of p‐TBK1. Indeed, wild‐type SMURF2 but not the C716A mutant efficiently promoted p‐TBK1 ubiquitination in HEK293T cells (Figure S7E, Supporting Information). Notably, the WW domain deletion mutant or the HECT domain deletion of SMURF2 failed to promote p‐TBK1 ubiquitination (Figure S7F, Supporting Information). Conversely, *SMURF2* deficiency reduced the ubiquitination of TBK1 (Figure S7G, Supporting Information). Neither *SMURF2* deficiency nor overexpression affects the ubiquitination of TBK1 S172A mutant (Figure S7G,H, Supporting Information). We also observed remarkably impaired ubiquitination of p‐TBK1 in MKO iBMDMs, compared to that in WT iBMDMs (Figure 7E). I*n vitro* ubiquitination assay further confirmed that SMURF2 directly ubiquitinated TBK1 (Figure 7F). These results strongly indicated that SMURF2 mediated the degradation of p‐TBK1.

To determine the ubiquitin chain type of SMURF2‐mediated p‐TBK1 ubiquitination, we transfected HEK293T cells with SMURF2‐ and TBK1‐expressing vectors in the presence of constructs expressing wild‐type ubiquitin (HA‐Ub) or its mutants. Western blot results revealed that SMURF2 mainly promoted Lys‐27 (K27‐Only, K27O)‐linked polyubiquitination of TBK1, and to a lesser extent, Lys‐29‐linked polyubiquitination (Figure 7G). Only the mutation of Lys‐27 to Arg (K27R) completely abrogated the poly‐ubiquitination effect of SMURF2 on TBK1 (Figure S7I, Supporting Information). In summary, these findings indicate that SMURF2 directly mediates Lys‐27‐linked ubiquitination of p‐TBK1 through its HECT domain, leading to the lysosomal degradation of p‐TBK1.

## 3 Discussion

We here showed a protective role of SMURF2 in autoimmune diseases. As a NEDD4 family E3 ligase, SMURF2 mediated K27 ubiquitination of p‐TBK1 and its degradation, which inhibits CSF1R signaling‐triggered macrophage proliferation, thereby restraining the autoimmune inflammation. However, bacterial invasion resulting from increased intestinal mucosal permeability suppresses SMURF2 expression in tissue‐resident macrophages (TRMs). This leads to overactivation of TBK1 and subsequent excessive TRM proliferation, ultimately exacerbating autoimmune inflammation. (Figure S7J, Supporting Information). We also demonstrated that the impaired expression of *Smurf2* in macrophages was related to the progression of autoimmune diseases in humans and mice.

It is well known that increased intestinal mucosal permeability during colitis leads to the invasion of bacteria into the colon lamina propria (CLP).^[^ ^44^ ^]^ We here demonstrated that invaded microbes released PAMPs to downregulate SMURF2 expression in colonic macrophages, and this process may involve epigenetic modification of histone H3. However, the underlying precise mechanism by which PAMPs downregulate SMURF2 expression remains to be further explored. In addition, we observed a notable reduction of SMURF2 expression in colonic epithelial cells of IBD patients and DSS‐treated mice. The regulatory role of SMURF2 in intestinal epithelial cell death and regeneration during colitis is still under investigation, and we are working to uncover the underlying mechanisms.

Macrophages, especially TRMs, play a key role in sensing pathogen invasion and responding to tissue injury. Dysregulation and dysfunction of TRMs are closely linked to autoimmune diseases.^[^ ^14^ ^]^ Microglial proliferation is a hallmark of EAE and is associated with disease progression in MS.^[^ ^45^ ^]^ In our study, loss of SMURF2 notably exacerbates the proliferation of TRMs and the development of autoimmune disease. TBK1 activation has been implicated in the proliferation of diverse tumor cells.^[^ ^38^ ^]^ Our findings demonstrated that TBK1 activation was essential for M‐CSF‐induced macrophage proliferation, while SMURF2 inhibited the proliferation of macrophages via targeting p‐TBK1.

As an anti‐inflammatory compound, the TBK1/IKKε‐specific inhibitor, ALX, mitigates chronic inflammation in EAE,^[^ ^40^ ^]^ Metabolic‐Associated Fatty Liver Disease (MAFLD), and obesity‐related metabolic dysfunction^[^ ^39^ ^]^ and has been used for the treatment of aphthous ulcers.^[^ ^46^ ^]^ Our investigation revealed that ALX administration exhibited minimal effects in DSS‐treated WT mice, but significantly alleviated the inflammatory response in *Smurf2* MKO mice. Importantly, it eliminated the differences in the progression of colitis and EAE between WT and MKO mice, indicating *Smurf2* deficiency accelerates autoimmune diseases via augmenting the level of p‐TBK1 in macrophages.

A recent paper reported that ALX exacerbates DSS‐induced colitis, in which mice were treated with a high dose of DSS (3.5%) and received 50 mg kg^−1^ ALX by gavage every 2 days.^[^ ^47^ ^]^ In our study, we pre‐treated mice with ALX by gavage one day prior, followed by feeding with 2.5% DSS and administering 25 mg kg^−1^ ALX by gavage at the same interval. These variations in experimental strategies could account for the inconsistency between our findings and those reported in the aforementioned study. Additionally, ALX's effects on colitis may be influenced by the diverse microbiome on account of different feeding environments.

We here demonstrated SMURF2 specifically mediated the degradation of p‐TBK1 and restrained M‐CSF‐induced proliferation of macrophages. However, SMURF2 deletion does not affect LPS‐induced TBK1/NF‐κB/MAPK activation and the expression of proinflammatory factors including *Il‐1β, Il‐6*, and *Tnf‐α* (Figure S8A,B, Supporting Information). A CO‐IP assay revealed that LPS induced a much weaker interaction between SMURF2 and p‐TBK1 than M‐CSF treatment, suggesting that SMURF2 exerts distinct regulatory effects on p‐TBK1 levels in response to different stimuli (Figure S8C, Supporting Information).

A significant reduction in SMURF2 expression was observed in peripheral blood mononuclear cells (PBMCs) from individuals with various macrophage dysfunction‐related autoimmune diseases,^[^ ^48^ ^]^ including juvenile idiopathic arthritis, adult‐onset Still's disease, systemic lupus erythematosus, and both primary and secondary autoimmune arthritis (Figure S8D–G, Supporting Information). These findings indicate that macrophage SMURF2 may exert a broad inhibitory role in autoimmune inflammation. Further investigation into the precise role of SMURF2 in these autoimmune diseases is warranted.

In conclusion, our study has unveiled the pivotal role of SMURF2 in restricting autoimmune inflammation via mediating ubiquitination of p‐TBK1 and inhibiting TRM proliferation. Given that the expression of SMURF2 in macrophages is impaired in autoimmune diseases and inversely correlates with disease progression, SMURF2 could be a potential therapeutic target for inflammatory disorders.

## 4 Experimental Section

### Sex as a Biological Variable

The study utilized both male and female biopsies from humans and tissue from mice, as sex was not considered a biological variable.

### Animals

*Smurf2^f/f^* (ES Cell Clone ID: EPD0424_6_H08) mice were acquired from MRC Harwell, UK. *Cx3cr1^Cre‐ERT^* (Stock Number: 02 1160) mice were provided by Professor Xiao Shen (Zhejiang University), and *Rosa26^tdTomato^* (Stock No: 0 07914) mice were supplied by Professor Chong Liu (Zhejiang University). Smurf2 myeloid‐specific knockout mice (MKO) were generated by crossing *Smurf2^f/f^* and *Lyz2‐iCre* (Strain NO. T003822, GemPharmatech, Nanjing, China). To generate microglia‐specific *Smurf2* conditional knockout mice, *Smurf2^f/f^* mice were crossed with *CX3CR1^Cre‐ERT^* mice. The CX3CR1^Cre‐ERT^; *Smurf2^f/f^* mice were intraperitoneally (i.p) injected with 3 mg tamoxifen dissolved in 200 µL corn oil for five consecutive days to induce the expression of Cre recombinase.^[^ ^14^, ^49^ ^]^ Six weeks later. The tamoxifen‐treated mice were used as microglia‐specific conditional *Smurf2* KO mice for the EAE study. Notably, CX3CR1^Cre+^ expressing blood cells, such as monocytes, are short‐lived^[^ ^50^, ^51^ ^]^ and are therefore replaced by their Cre‐negative monocyte progeny. To verify the specificity of *Smurf2* deletion in microglia, microglia were isolated from tamoxifen‐exposed mice using Percoll gradient centrifugation. Western blot analysis confirmed specific *Smurf2* knockout in microglia but not in bone marrow‐derived macrophages (BMDMs). All mice were housed in specific pathogen‐free (SPF) conditions at the Laboratory Animal Center of Zhejiang University, and all animal experiments strictly adhered to Institutional Animal Care and Use Committee guidelines at the School of Medicine, Zhejiang University.

### Human Subjects

Human paraffin‐embedded colon sections from IBD patients or normal control colon sections were obtained from the Department of Pathology, First Affiliated Hospital, Zhejiang University. Normal control colon sections consisted of healthy tissue from the resection edges of tumor biopsies that appeared to be healthy at the histological level. The basic information of the patients is summarized in the online supplemental Table S1 (Supporting Information).

### DSS‐Induced Colitis

For experimental colitis induction, WT and MKO or cKO mice were treated with 2.5% DSS in their drinking water for 5 days, followed by normal drinking water until the end of the experiment on day 9 or 12. During the experiment, body weights, stool, and bleeding were monitored daily to assess DAI. The DAI is the combined score of weight loss compared with initial weight, stool consistency, and bleeding scores were determined as previous study,^[^ ^31^ ^]^ DAI scores = body weight loss scores+ bleeding scores+ stool scores.

### Induction and Assessment of EAE

Acute EAE was induced and assessed as previously described.^[^ ^52^, ^53^ ^]^ Briefly, acute EAE was induced by subcutaneous immunization with 300 µg of the MOG_35‐55_ peptide in CFA containing 5 mg mL^−1^ heat‐killed H37Ra strain of Mycobacterium tuberculosis (Chondrex, Inc) in the back region and both sides of the vertebrae. And the immunized mice were i.v. injected with pertussis toxin (List Biological Laboratories, Inc.) at a dose of 250 ng per mouse in PBS on the day of immunization and once more 48 h after the first injection. The clinical score was performed in a double‐blinded manner. Mice were examined every 2–3 days for disease symptoms and were double‐blinded and scored for disease severity using the EAE scoring rulers: 0, no clinical signs; 1, limp tail; 2, paraparesis (weakness and incomplete paralysis of one or two hind limbs); 3, paraplegia (complete paralysis of two hind limbs); 4, paraplegia with fore limb weakness or paralysis; and 5, moribund state or death.

### Primary Cells and cell Lines

BMDMs were harvested and differentiated from bone marrow cells of mice in DMEM supplemented with 10% fetal bovine serum (FBS), 1% penicillin/streptomycin (P/S), and 2% M‐CSF conditioned medium or 20 ng mL^−1^ M‐CSF for 7 days. To generate iBMDMs, WT and MKO BMDMs were infected and immortalized by the J2 virus produced from the GG2EE cell line,^[^ ^54^ ^]^ and cultured in RPMI1640 (10 mM HEPES pH 7.8, 10% heat‐inactivated FBS, 1% P/S). Conditioned medium containing M‐CSF was collected from the supernatant of L929 cells (Gifted by Ma Feng, Suzhou Institute of Systems Medicine, China). HEK293T and RAW264.7 were obtained from the American Type Culture Collection (ATCC, Manassas, VA) and cultured in DMEM supplemented with 10% FBS and 1% P/S. For all experiments, cells were plated overnight, and the medium was replaced before stimulation, infection, or transfection. SMURF2‐overexpressed BMDMs were constructed using lentiviruses packaged in HEK293T cells that were transfected with pSPAX2, pMD2.G, and pHAGE‐Flag‐SMURF2/C716A‐zsGreen. SMURF2‐overexpressing RAW264.7 cells were generated by transfecting pXN‐Flag‐SMURF2 into RAW264.7, following selection with 5 µg mL^−1^ puromycin at 24 h post‐transfection. The selected cells were cultured in the medium with 0.5 µg mL^−1^ puromycin.

### Statistical Analysis

Statistical analysis was performed using GraphPad Prism 9 software. Data are presented as mean ± SEM. Statistical significance between two experimental groups was calculated using an unpaired two‐tailed Student's *t*‐test. The statistical significance of disease progression or cell proliferation was calculated using a two‐way analysis of variance (ANOVA) test. *P* < 0.05 was considered statistically significant (*P < 0.05, **P < 0.01, ***P < 0.001, ns, Non‐significant, *P* > 0.05).

### Study Approval

Written patient consent was provided, and ethics approval for human samples was granted by the Medical Ethics Committee of Zhejiang University School of Medicine (Ethics approval number: 2021‐005, 20210125–30, IIT20240689B‐R1) for harvesting human tissues. All animal experiments were approved by ZJU‐Laboratory Animal Welfare and Ethics Review Committee, Zhejiang University, China (Ethics approval: 202118445, ZJU20240482.).

## Conflict of Interest

The authors declare no conflict of interest.

## Author Contributions

X.A. and J.L. contributed equally to this work. X.A., W.L. and X.W. conceived and designed the study. J.L. provided patients samples and expert histopathological analysis. X.A., L.W., C.L. and Z.J. performed experiments. X.A. and X.W. analyzed the data and wrote the paper. M.J. supplied reagents, offered experimental advice and assisted in revising the manuscript to enhance its textual quality and English expression. Y.Y. helped revise the manuscript.

## Supporting information



Supporting Information

Supplemental Table 1

## Data Availability

The data is available in GEO database (GSE307224).
