# Supplementary material for: Tissue‐Resident Macrophage‐Derived E3 Ligase SMURF2 Restricts Autoimmune Inflammation by Mediating the Degradation of p‐TBK1
Source: Adv Sci (Weinh). 2025 Nov 21;13(7):e04930. doi: 10.1002/advs.202504930 (PMC12866865; doi:10.1002/advs.202504930)
Supplement: Supplementary file 1 — Supporting Information [file ADVS-13-e04930-s002.pdf]

Tissue-resident macrophage-derived E3 ligase SMURF2 restricts autoimmune inflammation by mediating the degradation of p-TBK1

Xiang An[1,2#], Jun Li[3#], Lingling Wang[1], Chengyuan Li[1], Zhenpeng Jin[1], Yushi Yao[2], Minmin Jiang[4*], Wenlong Lin[5*] and Xiaojian Wang[1*]

[1]Institute of Immunology and Bone Marrow Transplantation Center of The First Affiliated Hospital, School of Medicine, Zhejiang University, Hangzhou, 310058, China.

[2]Liangzhu Laboratory, Zhejiang University Medical Center, 1369 West Wenyi Road, Hangzhou, 311121, China.

[3]Department of Pathology, the First Affiliated Hospital, School of Medicine, Zhejiang University, Hangzhou, 310003, China.

[4]Shulan International Medical College, Zhejiang Shuren University, Hangzhou, 310015, China.

[5]The Second Affiliated Hospital, Zhejiang University School of Medicine, Hangzhou, China.

[#] These authors contributed equally.

*Corresponding authors:

1. Supplementary methods

2. Supplementary Figures and legends

Figure S1, related to Figure 1;

Figure S2, related to Figure 2;

Figure S3, related to Figure 3;

Figure S4, related to Figure 4;

Figure S5, related to Figure 5;

Figure S6, related to Figure 6;

Figure S7, related to Figure 7;

Figure S8.

3. Supplementary tables

Supplementary Methods

Reagents and antibodies

DSS was purchased from MP Biomedicals (M.W. =36,000-50,000); MOG_{35-55} peptide (residues 35-55, Met-Glu-Val-Gly-Trp-Tyr-Arg-Ser-Pro-Phe-Ser-Arg-Val-Val-His-Leu-Tyr-Arg-Asn-Gly-Lys) was synthesized by Sangon Biotech (Shanghai). Pertussis toxin (NC9675592) was purchased from List Biological Laboratories. Mycobacterium tuberculosis H37Ra (231141) was purchased from BD. Incomplete Freund's adjuvant (F5506) was purchased from Sigma-Aldrich. Amlexanox (HY-B0713), Chloroquine (HY-17589A), Brefeldin A (BFA) (HY-16592) and Tamoxifen (HY-13757A) were purchased from Med Chem Express (MCE). MG132 (S2619) was purchased from Selleck. Mouse M-CSF (CB34) was purchased from Novoprotein, LPS (L2630) and PGN (77140) were purchased from Sigma-Aldrich. Anti-Flag magnetic beads were purchased from GeneScript. Protein A/G and Anti-HA magnetic beads were purchased from MCE. PEI was purchased from Polyscience. Antibodies for SMURF2, Akt, p-Akt, TBK1, p-TBK1, p-JNK, JNK, p-ERK1/2, ERK1/2, p-p38, p38, IKKε, p-IKKε were purchased from Cell Signaling Technology. β-Actin, Anti-HA was purchased from Origene. Anti-Myc, HRP-anti-Rabbit, and AF488-anti-Rabbit were purchased from Proteintech. SMURF2 (dilution: 1:300) and Ubiquitin (dilution: 1:300) antibodies for Co-IP were purchased from Abclonal.

Plasmids

pHAGE-Flag-SMURF2 and pHAGE-Flag-SMURF2-C716A plasmids were generously provided by Professor Weiguo Zou (Chinese Academy of Sciences). SMURF2 was subcloned into the pLVX-puro, pcDNA3.1-HA-His, or pHAGE-IRES-ZsGreen vectors. Flag-TBK1-his and HA-TBK1 were kind gifts from Professor Pinglong Xu (Zhejiang University). Myc-SMURF2 deletion mutants were subcloned into the pLVX-puro vector.

Immunohistochemistry (IHC) & Immunofluorescence (IF) staining and score

Human colonic specimens from patients with IBD and normal control colon sections were immunohistochemically stained with anti-SMURF2 antibodies. The IHC samples were evaluated independently by three researchers (including one

pathologist). The IHC staining scores of SMURF2 were based on the positive area (0-3 score) and intensity score (0-4 score) of IHC staining. The SMURF2 intensity was then calculated by multiplying the positive area score and the intensity score.

For immunofluorescence staining, Samples were observed with FV1000-Olympus Confocal Microscopy. Fluorescence data were generated from three randomly captured digitized images using Olympus Olyvia software. For $Ki67^+$ cell analysis, the number of $CX3CR1^+Ki67^+$ or $F4/80^+Ki67^+$ double-positive cells from each case was calculated. The percentage of $Ki67^+$ cells in $CX3CR1^+$ or $F4/80^+$ macrophages was calculated by determining the proportion of $Ki67^+$ cells within these macrophage populations.

Single-cell RNA-Seq analysis

Single-cell data used in this study were acquired from the Single Cell Portal (SCP259). Data processing, including batch correction, doublet removal, gene annotation, and cell clustering, was performed as previously described [1]. After that, the Seurat R package (version 2.3.2) was used to normalize expression values for total unique molecular identifier counts per cell. The statistical significance was assessed using Kruskal-Wallis test.

Antibiotic treatment and fecal microbiota transplantation

Antibiotics were given through drinking water. For broad-spectrum antibiotic treatment, 0.5 mg/ml of vancomycin, 1 mg/ml of neomycin, 1 mg/ml of metronidazole and 1 mg/ml of ampicillin were added to drinking water containing Splenda (4 g/L), additionally, Amphotericin-B was either directly added to drinking water to prevent fungal overgrowth in the antibiotic-treated animals [2]. For FMT experiments, 200 mg of pooled fecal pellets from WT mice were homogenized with sterile silica beads in 1 mL PBS at 45 Hz for 1 min and filtered with 70-μm strainers. Abx-treated WT mice were subjected to gavage with 200 μl filtered stool homogenates twice with a 48 h interval.

Histological score

For histological analysis, paraffin-embedded sections (4 mm thick) were subjected to H&E staining. Histological scores were determined blindly based on the previously

described [3]. Briefly, 0=normal; 1=moderate mucosal inflammation without erosion or ulcer; 2=severe mucosal inflammation with erosion; 3=severe mucosal inflammation with ulcer (<1 mm); and 4=severe mucosal inflammation with ulcer (>1 mm).

Isolation and analysis of immune cells from the colon or CNS

Isolation of intestinal epithelial cells or colonic lamina propria cells was performed with an established method reported previously [3]. Briefly, after removing extra-intestinal fat tissue and blood vessel, colons were flushed of their luminal contents with cold PBS, opened longitudinally, and then incubated with PBS containing 5% FBS, 3 mM EDTA (Sigma-Aldrich), and 1 mM DTT (Sigma-Aldrich) for 30 min with shaking at 250 rpm to isolate the epithelial cells. The remaining colon tissue was washed three times and further cut into smaller 1 mm^2 pieces and then digested by 1 mg/ml Collagenase IV (Sigma-Aldrich) and 0.1 mg/ml DNase I (Roche) at 37°C with shaking for 60 min at 37°C. The Single-cell suspensions were obtained by grinding through a 70 μm cell strainer. After centrifugation at 600 g for 5 min, the fraction was resuspended in PBS containing 5% FBS for the following FACS analysis.

Isolation of intestinal epithelial cells or colonic lamina propria cells was performed with an established method reported previously [4]. Briefly, central nervous system tissues including spinal cords and brains, were homogenized in ice-cold tissue grinders, filtered through a 70 μm cell strainer (Falcon), and the cells were collected by centrifugation at 160 g for 5 min at 4°C. Cells were re-suspended in 10 ml of 35% Percoll (GE) and centrifuged onto a 5 ml 70% Percoll cushion in 15 ml tubes at 280 g for 25 min with a low accelerating or breaking speed. Cells at the 35-70% interface were collected and subjected to flow cytometry.

The colonic and CNS immune cells were prepared by using the abovementioned protocol, which was then applied for FACS sorting by using specific antibodies against related cell markers. The cells were analyzed with a NovoCyte Flow Cytometer (ACEA) or a BD Fortessa. Flow cytometric analysis was performed using FlowJo software.

Ex vivo colon organ culture

Colon was harvested and cut with scissor longitudinally. After washed with ice-cold PBS three times, 5 mm distal colon was cultured in 1 ml of RPMI 1640 complete medium for 24 h at 37°C. Cytokines in culture supernatants were measured by mouse IL-6 and TNF-α ELISA kits (eBioscience) and mouse M-CSF ELISA kits (J&L Biological) according to the manufacturers' instruction.

Real-time qRT-PCR

Total RNA was isolated by using TRIzol reagent (AG) and subjected to cDNA synthesis. qRT-PCR was performed in triplicate by using SYBR Green Supermix (AG). The expression of individual genes was calculated by a standard curve method and normalized to the expression of *Actb* or *GAPDH*. The gene-specific PCR primers are shown in the online supplemental Table S2.

***SMURF2* knockout HEK293T cell line generation**

Small guide RNAs (gRNA) target SMURF2 for knockout were designed and subcloned into a Lenti-CRISPR-V2 vector. After transfection, HEK293T cells were further screened using 1 μg/ml puromycin. The surviving cell was further amplified to get multiclonal cell lines. The KO cells were detected by WB analysis. The sequence for human SMURF2 gRNA sequence is shown in the online supplemental Table S3.

Colony formation and Proliferation assay

Bone marrow cells were mixed with methylcellulose (M3134) and IMDM (both from Stem Cell Technologies) according to the manufacturer's instructions in the presence of 10% FBS and M-CSF (20 ng/ml). Each methylcellulose dish contained 2×10^4 cells, and samples were plated in replicates. CFUs were recorded on day 3, day 5 and day 7 of cell culture. For proliferation assay, 1×10^4 BM cells were cultured in RMPI1640 medium in the presence of 10% FBS and M-CSF (20 ng/ml), The numbers of BMDMs were induced at various times.

Immunoprecipitation and immunoblot analyses.

For endogenous Co-IP assay, iBMDMs were pre-stimulated with M-CSF (50 ng/ml) for 30 min, then harvested and lysed in a lysis buffer. After centrifugation for 15 min

under 12,000 g at 4°C, the supernatants were collected and subjected to overnight incubation with anti-SMURF2 (1:300) or anti-pTBK1 (1:300) antibody and protein A/G magnetic beads. The beads were washed with lysis buffer three times, and the proteins were eluted by loading buffer for immunoblot assay. For exogenous CO-IP, Empty vector control (Null), SMURF2 (or its domain mutants) and TBK1 (or its S172 mutated form) plasmids were co-transfected into HEK293T cells, and the cell lysates were harvested and processed as described in endogenous co-IP. The Protein G magnetic beads were substituted by anti-Flag beads. For WB analysis, the cells were harvested and were lysed in lysis buffer (5% glycerol, 1% Nonidet P-40, 0.1% SDS, 5 mM EDTA, 100 mM NaCl, 20 mM Tris–HCl [pH8.0], 25 mM β-glycerophosphate, 1 mM phenylmethylsulfonyl fluoride, 1 mM sodium orthovanadate, 1 mM sodium fluoride, 1 μg/ml aprotinin, and 1 μg/ml leupeptin). After centrifugation, the cell lysates was subjected to SDS–PAGE for separation and subsequently, transferred onto nitrocellulose membranes and then for antibody detection.

Pull-down assay

Flag-TBK1 and HA-SMURF2 proteins were purified from HEK293T cells. TBK1 bound to anti-Flag beads were mixed with HA-SMURF2 or HA-GFP and incubated at 4°C for 4 h with gentle rotation. The beads were washed three times with cell lysis buffer. Then, bound proteins were extracted with loading buffer and analyzed by immunoblot.

Ubiquitination assay

Ubiquitination assays were performed as described previously [5]. For endogenous p-TBK1 ubiquitination assay, WT or SMURF2 Knockout iBMDMs were lysed and boiled for 10 min in lysis buffer supplemented with 1% SDS. After 5 min cooling in ice, the cell lysate was diluted ten times with lysis buffer, and then centrifugation for 10 min under 12,000 g at 4°C, the supernatants were collected and subjected to overnight incubation with anti-ubiquitin antibody (Abcam, USA) and protein A/G magnetic beads. After incubation, the beads were washed three times with cell lysis buffer and then eluted by loading buffer and subsequently, for SDS-PAGE separation. For exogenous p-TBK1 ubiquitination assay, HEK293T cells were transfected with

Flag-TBK1, Myc-SMURF2 and HA-Ubiquitin or its mutants, 36 h later, 20 μM (final concentration) of BFA was added to inhibit the function of proteasomes and 6 h later, cells were harvested and processed as described in endogenous ubiquitination assay. The Protein G magnetic beats were substituted by Anti-Flag beads.

RNA-Seq analysis

WT and MKO BMDMs were serum and M-CSF starved for 4 h followed by 50 ng/ml M-CSF stimulation for 12 h, these cells were subjected to HiSeq RNA-Seq performed by OE Biotech Co., Ltd (Shanghai, China). Transcriptomic reads from the RNA-Seq experiments were mapped to a reference genome (mm10) by using Bowtie. Gene expression levels were quantified by using the RSEM software package. Significant genes were defined by the p-value and false discovery rate of cutoff of 0.05 and fold changes $\geq$1.5. Differentially expressed genes were subsequently analyzed by using the DAVID bioinformatics platform and Ingenuity Pathway Analysis. The genes used for the GO term analyses are the up-and down-regulated genes in M-CSF-stimulated.

ChIP-Quantitative PCR assay

ChIP assays were performed according to the manufacturer's instructions with a Sonication ChIP Kit (Magnetic Beats) (Abclonal, RK20258). Briefly, iBMDMs were fixed by 1% formaldehyde. The cross-linked chromatin was sonicated in a water bath at 4°C using Bioruptor UCD-200 sonicator to obtain DNA fragments sized between 150 and 500 base pairs. Chromatin from 1×10^6 cells was used for each ChIP experiment. Antibodies against H3K4me3, H3K9me3, H3K27me3, or H3K36me3 were used. The DNA from cross-linking was purified for quantitative PCR analysis.

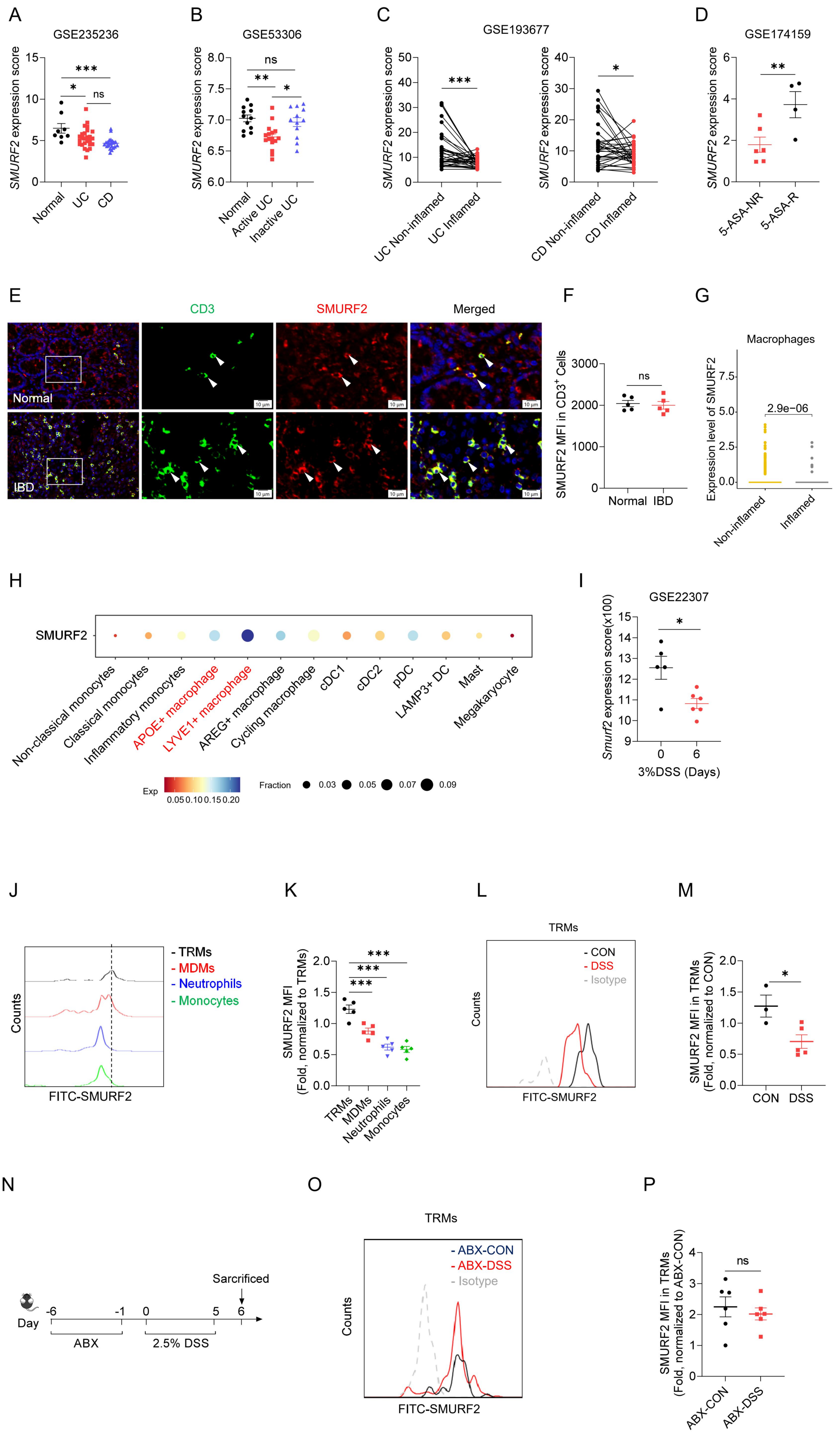
A
GSE235236
SMURF2 expression score
Normal
UC
CD
B
GSE53306
Active UC
Inactive UC
C
GSE193677
UC Non-inflamed
UC Inflamed
CD Non-inflamed
CD Inflamed
D
GSE174159
5-ASA-NR
5-ASA-R
E
CD3
SMURF2
Merged
Normal
IBD
F
SMURF2 MFI in CD3+ Cells
G
Macrophages
Expression level of SMURF2
2.9e−06
Non-inflamed
Inflamed
H
SMURF2
Non-classical monocytes
Classical monocytes
Inflammatory monocytes
APOE+ macrophage
LYVE1+ macrophage
AREG+ macrophage
Cycling macrophage
cDC1
cDC2
pDC
LAMP3+ DC
Mast
Megakaryocyte
Exp
Fraction
I
GSE22307
Smurf2 expression score(x100)
3%DSS (Days)
J
TRMs
MDMs
Neutrophils
Monocytes
Counts
FITC-SMURF2
K
SMURF2 MFI
(Fold, normalized to TRMs)
L
TRMs
CON
DSS
Isotype
M
SMURF2 MFI in TRMs
(Fold, normalized to CON)
N
Sarcrificed
Day
ABX
2.5% DSS
O
ABX-CON
ABX-DSS
P
SMURF2 MFI in TRMs
(Fold, normalized to ABX-CON)

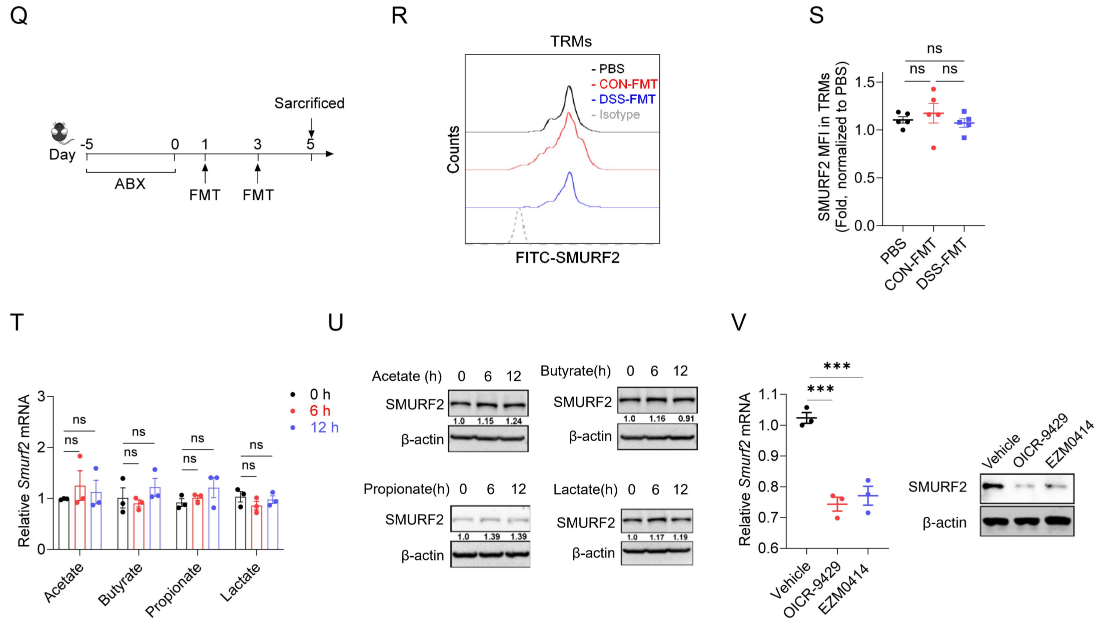

Figure S1. Reduced SMURF2 expression in inflamed intestinal tissues.

(A, B) Relative *SMURF2* expression in the colonic RNA-seq data of normal and IBD patients **(A)** or normal, active and inactive UC patients **(B)**.

(C) Relative *SMURF2* expression in the colonic RNA-seq data of paired non-inflamed and inflamed colonic tissue obtained from active IBD patients.

(D) Relative *SMURF2* expression in the colonic RNA-seq data of IBD patients treated with 5-aminosalicylic acid (5-ASA). NR, non-responder; R, responder.

(E) Representative SMURF2 and CD3 immunofluorescence staining of specimens obtained from IBD patients and healthy controls. Scale bars, 10 μm.

(F) MFI of SMURF2 in $CD3^+$ T cells as described in **E,** (n=5).

(G) Single-cell RNA-seq analysis of *SMURF2* expression in colonic macrophages within paired non-inflamed or inflamed colonic mucosa tissues (Single Cell Portal: SCP259). Each dot represents a macrophage.

(H) Single-cell RNA-seq analysis of the *SMURF2* expression profile in colonic by using scIBD database (Single Cell Portal: SCP259).

(I) Relative *Smurf2* expression in the colonic RNA-seq data of colon tissue from water or 3% DSS-fed mice.

(J) Representative flow cytometry analysis of SMURF2 expression in colonic TRMs, MDMs, neutrophils and monocytes.

(K) Flow cytometry analysis of SMURF2 MFI in myeloid cells as described in **L**.

(L) Representative flow cytometry analysis of SMURF2 expression in colonic TRMs from *Cx3cr1*$^{\text{CreERT2/+}}$: *R26*^{tdTomato} reporter mice treated with or without DSS on day 6 (DSS fed for 5 days).

(M) Flow cytometry analysis of SMURF2 MFI in myeloid cells as described in **N**.

(N-P) *Cx3cr1*$^{\text{CreERT2/+}}$: *R26*^{tdTomato} reporter mice were pretreated with Broad-spectrum antibiotics (ABX) for 5 days, followed by ABX or 2.5% DSS+ABX feeding. Mice was sacrificed on day 6 and followed by flow cytometry. Schematic diagram of ABX treatment **(N)**. Representative flow cytometry analysis of SMURF2 expression **(O)** and SMURF2 MFI in colonic TRM **(P)**, n=6 per group.

(Q-S) *C57BL6* mice were pretreated with Broad-spectrum antibiotics (ABX) for 5 days, followed by two times fecal microbiota transplantation (FMT) by using PBS or fecal samples from control mice (CON-FMT) or DSS-treated mice (DSS-FMT)**.** Schematic diagram of FMT treatment **(Q)**. Representative flow cytometry analysis of SMURF2 expression **(R)** and SMURF2 MFI in colonic TRM **(S)**, n=5 per group.

(T, U) RT-qPCR analysis **(T)** and Western blotting analysis **(U)** of SMURF2 expression in iBMDMs treated with Short-chain fatty acids (SCFAs, acetate, butyrate and propionate) or lactate for indicated times.

(V) RT-qPCR analysis (left) and Western blotting analysis of SMURF2 expression in iBMDMs treated with H3K4me3 inhibitor (OICR-9429, 20μM) and H3K36me3 inhibitor (EZM0414, 20μM) for 12 h.

Data are shown as mean ± SEM. Each dot represents a biological replicate (**A-D, F, G, K, M, P, S**) or a technical replicate **(T, V)**. Data are representative of at least two independent experiments **(F, G, J, L-W)**. *p<0.05, ** p<0.01, ***p<0.001, ns, Non-significant, P > 0.05. P values were calculated by 2-tailed Student's t test (**C, D, F, G, I, M, P**) or one-way ANOVA (**A, B, K, S, T, V**).

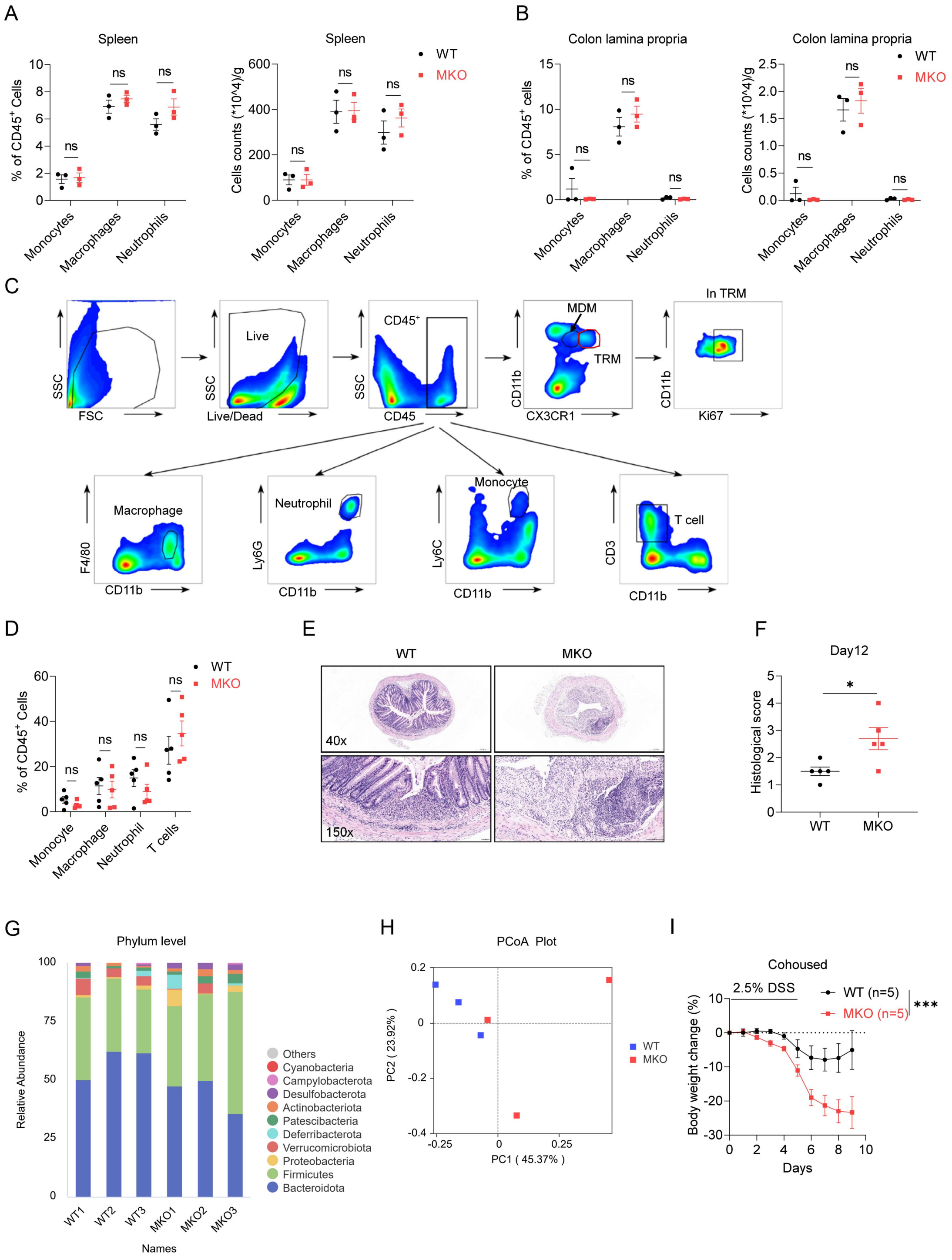
A
Spleen
% of CD45+ Cells
Monocytes
Macrophages
Neutrophils
ns
Spleen
Cells counts (*10^4)/g
WT
MKO
B
Colon lamina propria
% of CD45+ cells
Colon lamina propria
Cells counts (*10^4)/g
C
SSC
FSC
Live
Live/Dead
CD45+
CD45
MDM
TRM
CD11b
CX3CR1
In TRM
Ki67
Macrophage
F4/80
Neutrophil
Ly6G
Monocyte
Ly6C
T cell
CD3
D
% of CD45+ Cells
Monocyte
Macrophage
Neutrophil
T cells
E
WT
MKO
40x
150x
F
Day12
Histological score
*
G
Phylum level
Relative Abundance
Others
Cyanobacteria
Campylobacterota
Desulfobacterota
Actinobacteriota
Patescibacteria
Deferribacterota
Verrucomicrobiota
Proteobacteria
Firmicutes
Bacteroidota
WT1
WT2
WT3
MKO1
MKO2
MKO3
Names
H
PCoA Plot
PC2 (23.92%)
PC1 (45.37%)
I
Cohoused
2.5% DSS
WT (n=5)
MKO (n=5)
***
Body weight change (%)
Days

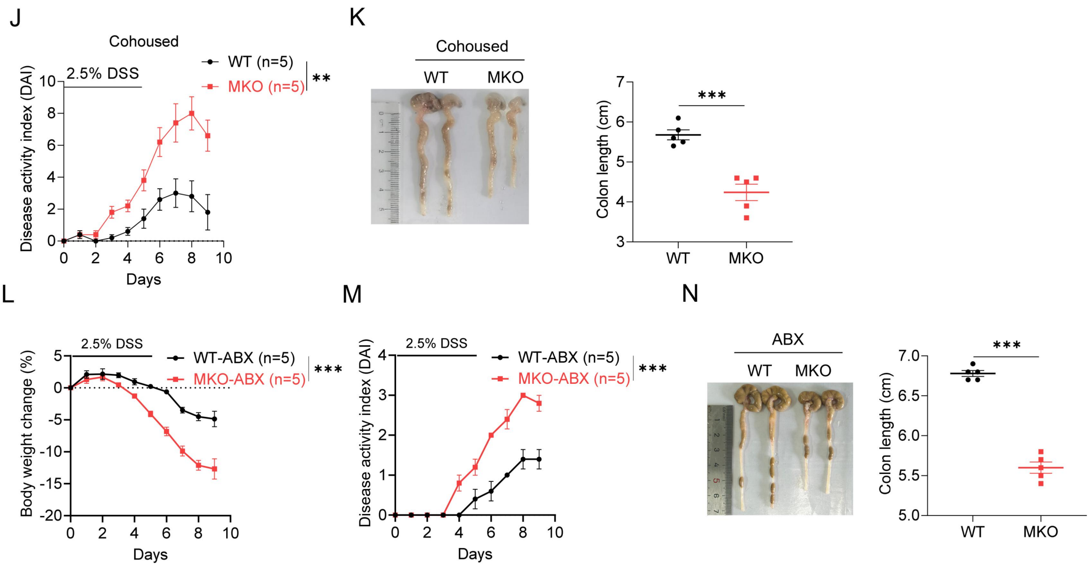

Figure S2. *Smurf2* deficiency in myeloid cells exacerbates experimental colitis.

(A, B) Spleen and colon lamina propria (CLP) of WT and MKO mice were harvested and digested to generate single cells. Quantified percentages (left) and cell numbers (right) of myeloid cells in the spleen **(A)** and CLP **(B)** were determined by flow cytometry. n=3 per group.

(C) Gating strategy for flow cytometry analysis of immune cells in the CLP.

(D) WT and MKO mice were administered 2.5% DSS for 5 days followed by 4 days water to induce acute colitis. Flow cytometry analysis of quantified percentages of myeloid cells in CLP from mice.

(E, F) WT and MKO mice were treated with 2.5% DSS for 5 days, and then fed with water for 7 days. Representative H&E staining **(E)** and histopathological score of colonic sections **(F)** from WT and MKO mice.

(G, H) Fecal samples were collected from WT and MKO mice for 16S rDNA gene sequencing. Alteration of microbial composition at phylum levels **(G).** The analysis of beta-diversity (Principal Co-ordinates Analysis: PCoA, based on unweighted UniFrac) in WT and MKO groups **(H).**

(I-K) WT mice and MKO mice were cohoused for 4 weeks before DSS treatment. After DSS treatment, changes in body weight **(I)**, and DAI **(J)** were assessed daily, gross morphology images and colon length **(K)** was measured on day 9.

(L-N) WT mice and MKO mice were pretreated with Broad-spectrum antibiotics

(ABX) for 5 days, followed by ABX or 2.5% DSS+ABX feeding. Changes in body weight **(L)**, and DAI **(M)** were assessed daily, gross morphology images and colon length was measured on day 9 **(N)**.

Data are shown as mean ± SEM. Each dot represents a biological replicate **(A, B, D, F, K, N)**, the n values also represent the number of biological replicates **(I, J, L, M)**. Data are representative of at least two independent experiments **(A, B, D-I)**. *$p<0.05$, ** $p<0.01$, ***$p<0.001$, ns, Non-significant, $P > 0.05$. P values were calculated by using two-way ANOVA **(I, J, L, M)** or 2-tailed Student’s t-tests **(A, B, D, F, K, N)**.

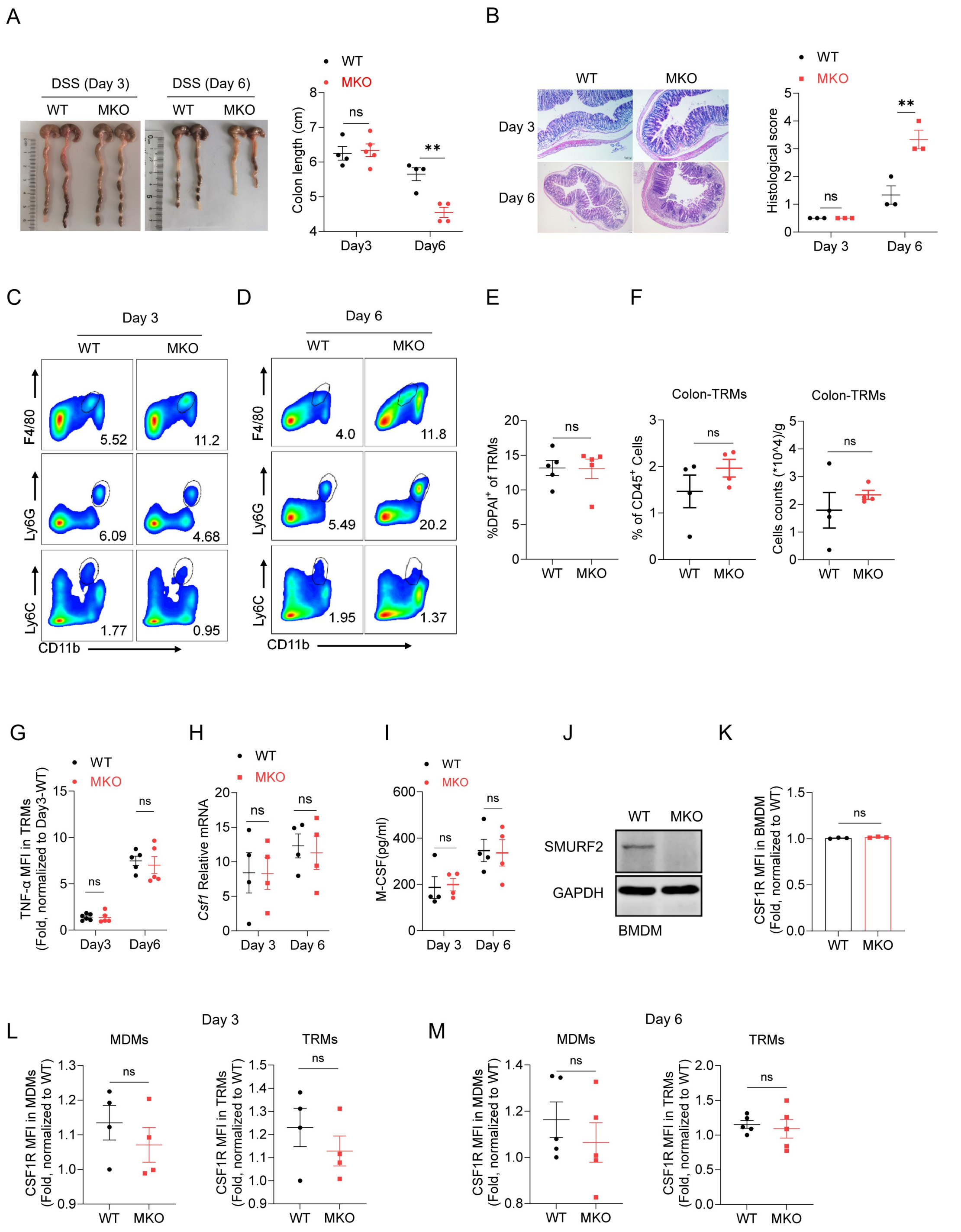

Figure S3. SMURF2 suppresses TRMs expansion in colitis.

(A-D) *Smurf2* WT and MKO mice were fed with 2.5% DSS and then sacrificed on day 3 or 6 (DSS fed for 5 days), followed by flow cytometry analysis of colon-infiltrated immune cells. Gross morphology images (left) and colon length (right) of WT and MKO mice at day 3 and day 6 **(A)**. **(B)** Colon samples from WT and MKO mice as described in **A** were collected and subjected to H&E staining and

histological score statistics (n=3 per group). Representative flow cytometry analysis of colonic myeloid cells of WT and MKO mice on day 3 **(C)** and day 6 **(D)** after DSS treatment.

(E) Flow cytometry analysis of the percentages of $DAPI^+$ cells in CLP TRMs from WT and MKO mice on day 3 after colitis induction.

(F) Colon samples from WT and MKO mice as were collected and subjected to flow cytometry analysis (n=4 per group) for the percentage of TRMs **(up)** and cell number of TRMs **(down).**

(G) Flow cytometry analysis of the TNF-α MFI in colonic TRMs from WT MKO mice as described in **A**.

(H, I) RT-qPCR **(H)** and ELISA **(I)** analysis of M-CSF (*Csf1*) expression in colon tissue from WT and MKO mice as described in **A**.

(J) Western blotting analysis of the expression of SMURF2 in BMDMs from WT and MKO mice.

(K) Flow cytometry analysis of the CSF1R MFI in WT and MKO BMDMs.

(L-M) Flow cytometry analysis of the CSF1R MFI in colonic MDMs and TRMs from WT and MKO mice on day 3 **(L)** and day 6 **(M)** after colitis induction.

Data are shown as mean ± SEM. All dot represents a biological replicate. Data are representative of at least two independent experiments. ** $p<0.01$, ns, Non-significant, $P > 0.05$. P values were calculated by using 2-tailed Student's t-tests.

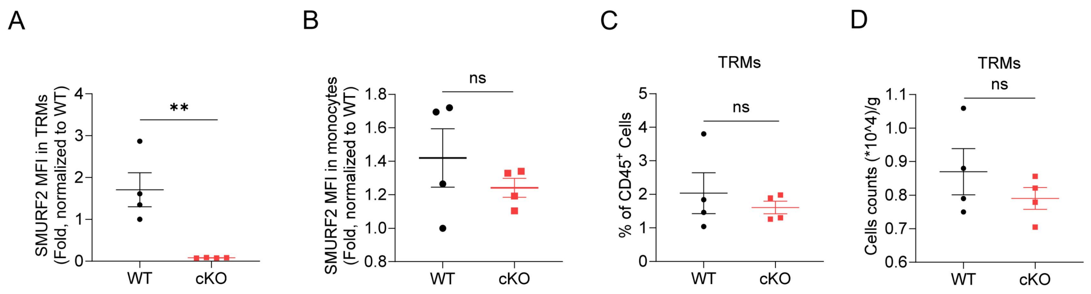

Figure S4. TRM-specific *Smurf2* deficiency exacerbates experimental colitis.

(A, B) Flow cytometry analysis of the SMURF2 MFI in colonic TRMs **(A)** and monocytes **(B)** from WT and cKO mice.

(C, D) Flow cytometry analysis of the percentages **(C)** and cell numbers (D) of colonic TRMs from WT and cKO mice.

Data are shown as mean ± SEM. All dot represents a biological replicate. Data are representative of at least two independent experiments. ** $p<0.01$, ns, Non-significant, $P > 0.05$. P values were calculated by using 2-tailed Student's t-tests.

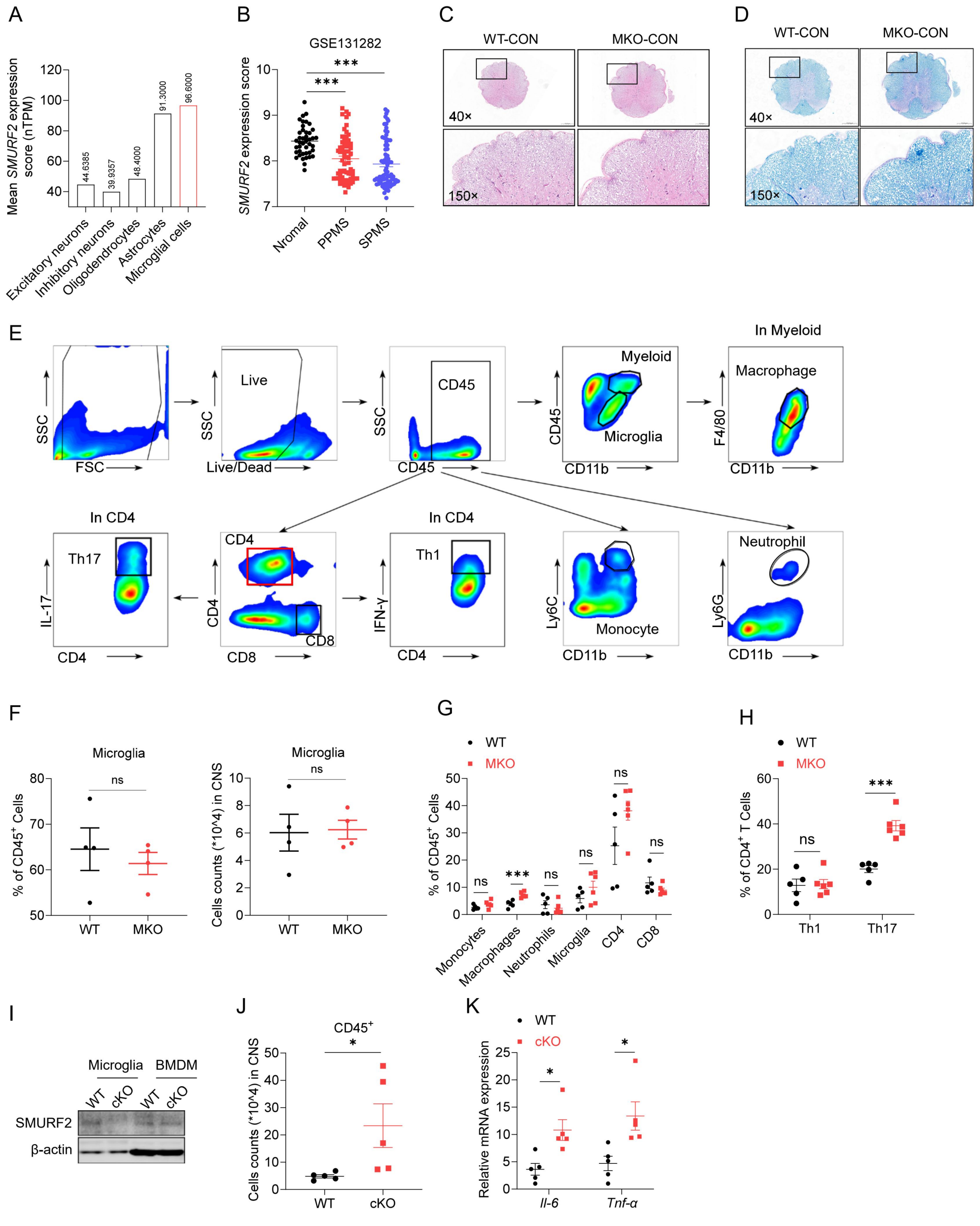

Figure S5. *Smurf2* deletion in microglia promotes the development of experimental autoimmune encephalomyelitis (EAE).

(A) The expression profile of *SMURF2* in brain cells in single-cell sequencing data from The Human Protein Atlas (HPA).

(B) Relative *SMURF2* expression in the RNA-seq data of grey matter obtained from normal control and multiple sclerosis patients Primary progressive MS (PPMS), Secondary progressive MS (SPMS).

(C, D) Representative H&E **(C)** and LFB staining **(D)** of the spinal cord from normal WT and MKO mice, n=3 per group.

(E) Gating strategy for analyzing immune cells in the CNS (Brain and spinal cord).

(F) Central nervous system (CNS) samples from WT and MKO mice were collected and subjected to flow cytometry analysis for the percentage of microglia **(left)** and cell number of microglia **(right).**

(G, H) WT and MKO mice were immunized with MOG_{35-55} for 20 days to induce EAE. Flow cytometry analysis of the quantified percentages of myeloid cells, microglia and T cells **(G)**; Th1 and Th17 cells **(H)** in the CNS from WT and cKO mice.

(I) Western blotting analysis of SMURF2 expression in microglia and BMDMs from WT and cKO mice.

(J) Flow cytometry analysis of the cell numbers of $CD45^+$ immune cells in the CNS from WT and cKO mice on day 20 of EAE induction.

(K) RT-qPCR analysis of *Il-6, Tnf-α* expression in the CNS from WT and cKO mice on day 20 of EAE induction.

Data are shown as mean ± SEM. Each dot represents a biological replicate **(B, F, G, H, J, K)**. Data are representative of at least two independent experiments **(C, D, F-J)**. *p<0.05, ***p<0.001, ns, Non-significant, P > 0.05. P values were calculated by using one-way ANOVA **(B)** or 2-tailed Student's t-tests **(F, G, H, I, J)**.

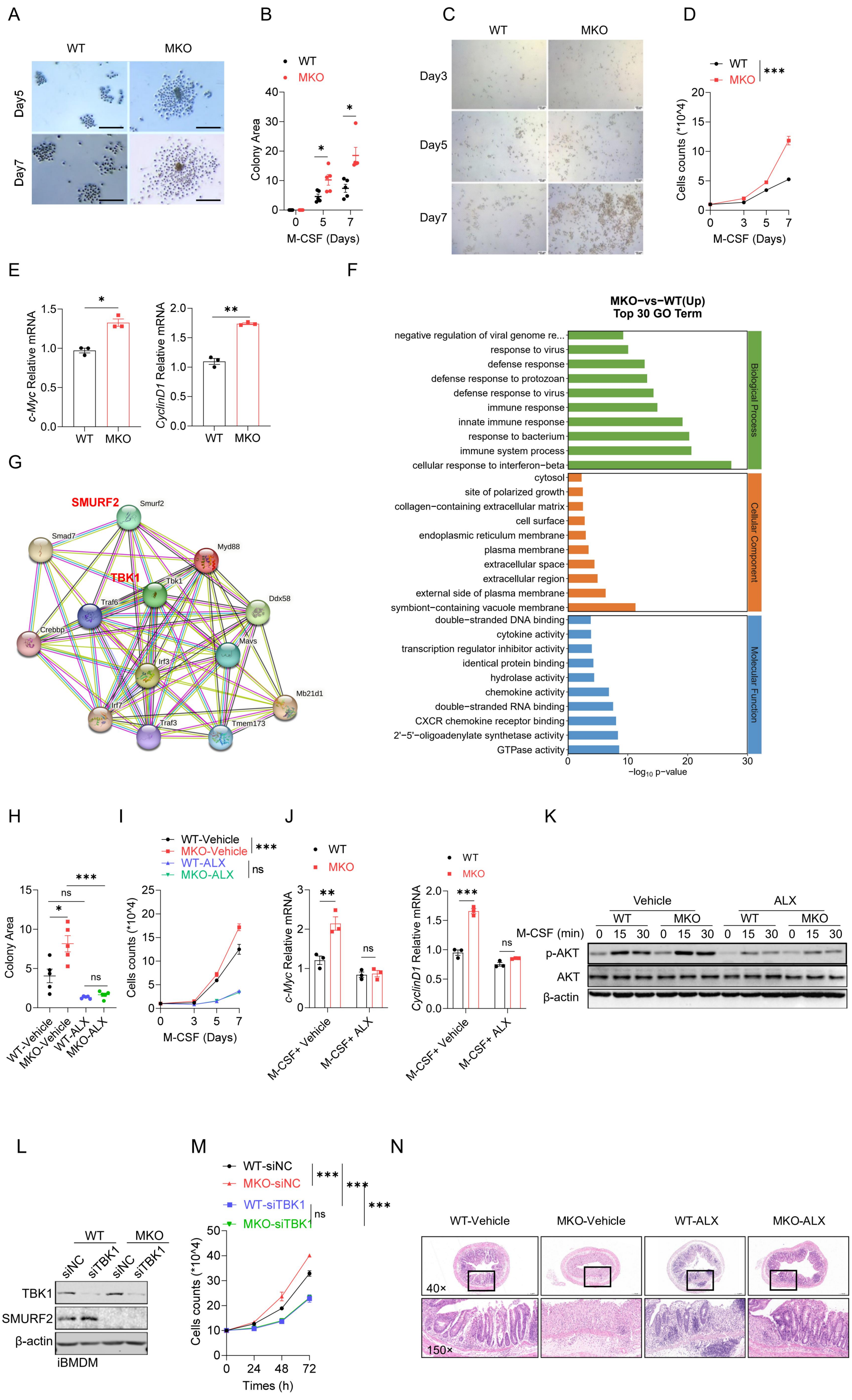
A
WT
MKO
Day5
Day7
B
WT
MKO
Colony Area
M-CSF (Days)
C
WT
MKO
Day3
Day5
Day7
D
WT
MKO
***
Cells counts (*10^4)
M-CSF (Days)
E
c-Myc Relative mRNA
CyclinD1 Relative mRNA
WT MKO
F
MKO−vs−WT(Up)
Top 30 GO Term
negative regulation of viral genome re...
response to virus
defense response
defense response to protozoan
defense response to virus
immune response
innate immune response
response to bacterium
immune system process
cellular response to interferon−beta
cytosol
site of polarized growth
collagen−containing extracellular matrix
cell surface
endoplasmic reticulum membrane
plasma membrane
extracellular space
extracellular region
external side of plasma membrane
symbiont−containing vacuole membrane
double−stranded DNA binding
cytokine activity
transcription regulator inhibitor activity
identical protein binding
hydrolase activity
chemokine activity
double−stranded RNA binding
CXCR chemokine receptor binding
2'−5'−oligoadenylate synthetase activity
GTPase activity
Biological Process
Cellular Component
Molecular Function
−log10 p−value
G
SMURF2
Smurf2
Smad7
Myd88
TBK1
Tbk1
Traf6
Ddx58
Crebbp
Mavs
Irf3
Mb21d1
Irf7
Traf3
Tmem173
H
Colony Area
WT-Vehicle
MKO-Vehicle
WT-ALX
MKO-ALX
I
WT-Vehicle
MKO-Vehicle
WT-ALX
MKO-ALX
Cells counts (*10^4)
M-CSF (Days)
J
WT
MKO
c-Myc Relative mRNA
CyclinD1 Relative mRNA
M-CSF+ Vehicle
M-CSF+ ALX
K
Vehicle
ALX
WT
MKO
M-CSF (min)
p-AKT
AKT
β-actin
L
WT
MKO
siNC
siTBK1
TBK1
SMURF2
β-actin
iBMDM
M
WT-siNC
MKO-siNC
WT-siTBK1
MKO-siTBK1
Cells counts (*10^4)
Times (h)
N
WT-Vehicle
MKO-Vehicle
WT-ALX
MKO-ALX
40×
150×

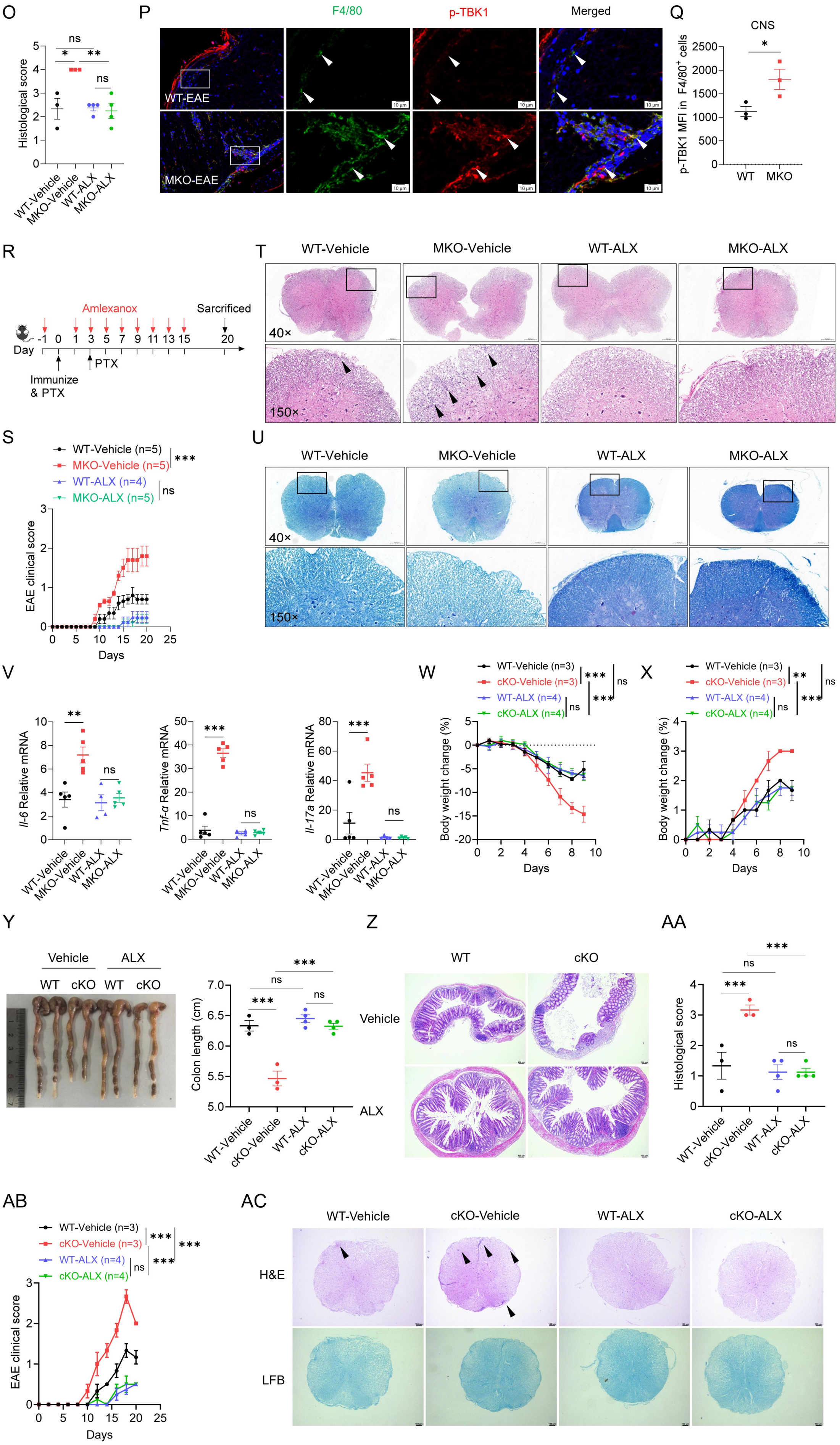

O
ns
*
**
ns
Histological score
WT-Vehicle
MKO-Vehicle
WT-ALX
MKO-ALX
P
F4/80
p-TBK1
Merged
WT-EAE
MKO-EAE
Q
CNS
*
p-TBK1 MFI in F4/80+ cells
WT
MKO
R
Amlexanox
Sarcrificed
Day
-1 0 1 3 5 7 9 11 13 15 20
Immunize & PTX
PTX
T
WT-Vehicle
MKO-Vehicle
WT-ALX
MKO-ALX
40×
150×
S
WT-Vehicle (n=5)
MKO-Vehicle (n=5)
WT-ALX (n=4)
MKO-ALX (n=5)
***
ns
EAE clinical score
Days
U
WT-Vehicle
MKO-Vehicle
WT-ALX
MKO-ALX
40×
150×
V
Il-6 Relative mRNA
Tnf-α Relative mRNA
Il-17a Relative mRNA
**
***
***
ns
WT-Vehicle
MKO-Vehicle
WT-ALX
MKO-ALX
W
WT-Vehicle (n=3)
cKO-Vehicle (n=3)
WT-ALX (n=4)
cKO-ALX (n=4)
***
***
ns
ns
Body weight change (%)
Days
X
WT-Vehicle (n=3)
cKO-Vehicle (n=3)
WT-ALX (n=4)
cKO-ALX (n=4)
**
***
ns
ns
Body weight change (%)
Days
Y
Vehicle
ALX
WT cKO WT cKO
Colon length (cm)
***
ns
***
ns
WT-Vehicle
cKO-Vehicle
WT-ALX
cKO-ALX
Z
WT
cKO
Vehicle
ALX
AA
***
ns
***
ns
Histological score
WT-Vehicle
cKO-Vehicle
WT-ALX
cKO-ALX
AB
WT-Vehicle (n=3)
cKO-Vehicle (n=3)
WT-ALX (n=4)
cKO-ALX (n=4)
***
***
***
ns
EAE clinical score
Days
AC
WT-Vehicle
cKO-Vehicle
WT-ALX
cKO-ALX
H&E
LFB

Figure S6. *Smurf2* deficiency promotes macrophage proliferation and aggravates autoimmune inflammation in a TBK1-dependent manner.

(A, B) *Smurf2* WT and MKO bone marrow cells were cultured with M-CSF (20 ng/ml) and colony formation was assayed on day 5 and day 7. Data are presented as representative colony pictures **(A)** and colony area measured by ImageJ **(B)**. Each dot indicates a colony, n=5 per group.

(C, D) *Smurf2* WT and MKO bone marrow cells were cultured with M-CSF and cell number was counted at day 3, 5 and 7 during culture, data are presented as representative pictures **(C)** and cell number **(D)**.

(E) RT-qPCR analysis of *c-Myc* and *CyclinD1* expression in WT and MKO BMC cultured with M-CSF (20 ng/ml) for 5 days.

(F, G) *Smurf2* WT and MKO BMDMs were M-CSF starved for 4 h, followed by 12 h M-CSF (50 ng/ml) stimulation for transcriptome RNA-seq assay. Data is presented as GO Enrichment **(F)** and STRING analysis **(G)**.

(H) WT and MKO bone marrow cells were cultured with M-CSF with or without 50 μM Amlexanox (ALX) and the colony area was assayed on day 7.

(I) WT and MKO bone marrow cells were treated the same as described in **H**, cell number was counted at day 3, 5 and 7 during culture.

(J) RT-qPCR analysis of *c-Myc* and *CyclinD1* expression in WT and MKO BMC treated as described in **H**.

(K) Western blotting analysis of AKT activation in WT and MKO BMDMs which were pretreated with or without 150 μM ALX for 1 h, followed by M-CSF (50 ng/ml) stimulation for the indicated times.

(L) Western blotting analysis of the expression of TBK1, SMURF2 in WT and MKO iBMDMs transfected with siRNA mimic (siNC) or siTBK1.

(M) WT and MKO iBMDMs were transfected with siRNA mimic (siNC) or siTBK1, followed by cultured with M-CSF and cell number was counted at 24, 48, 72 h during culture, data are presented as cell number.

(N, O) Representative H&E staining of the colon **(N)** and histological score **(O)** of mice from **6F**. n=3-4 per group.

(P) Representative p-TBK1 and F4/80 IF staining in mice spinal cord sections from WT and MKO-EAE mice. Scale bars, 10 μm.

(Q) The MFI of p-TBK1 in $F4/80^+$ macrophages as described in **P**.

(R-V) Age and gender-matched WT and MKO mice were orally administrated TBK1 inhibitor ALX at a dose of 25 mg/kg every 2 days. WT and MKO mice were immunized with MOG_{35-55} for 20 days to induce EAE. Schematic diagram of ALX treatment **(R)**. Mean clinical scores of EAE mice **(S)**. Representative H&E staining **(T)** and LFB staining **(T)** of the spinal cord, the mRNA level of *Il-6*, *Tnf-α* and *Il-17a* in CNS tissue **(U)** from EAE mice. n= 4-5 per group.

(W-AA) WT and cKO mice were treated as **6F**. Body weight change **(W)**, DAI **(X)** were assessed daily, gross morphology images of colon and colon length **(Y)** . H&E staining **(Z)** and histological score was measured on day 9 **(AA).**

(AB-AC) WT and cKO mice were treated as **S6R**. Mean clinical scores of EAE mice **(AB)**. Representative H&E staining and LFB staining **(AC)** of the spinal cord from EAE mice.

Data are shown as mean ± SEM. Each dot represents a biological replicate **(B, D, H, I, M, O, Q, V, Y, AA)** or a technical replicate **(E, J)**, the n values also represent the number of biological replicates **(S, W, X, AB)**. Data are representative of at least two independent experiments **(A-E, H-V)**. *p<0.05, ** p<0.01, ***p<0.001, ns, Non-significant, P > 0.05. P values were calculated by using two-way ANOVA **(D, I, M, S, AB)** or 2-tailed Student's t-tests **(B, E, J, Q)** or one-way ANOVA **(H, O, V, Y, AA)**.

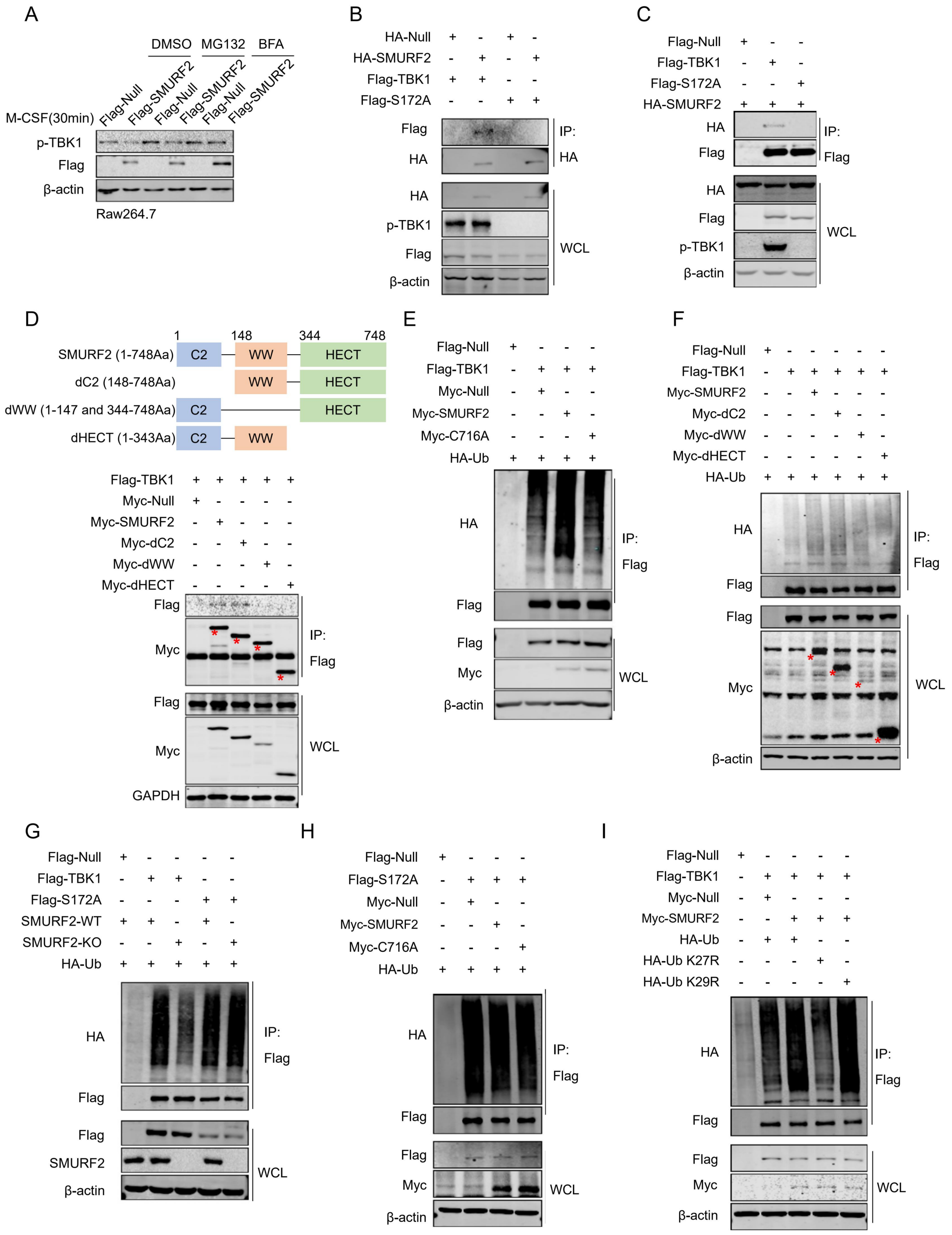
A
DMSO
MG132
BFA
M-CSF(30min)
Flag-Null
Flag-SMURF2
Flag-Null
Flag-SMURF2
Flag-Null
Flag-SMURF2
p-TBK1
Flag
β-actin
Raw264.7
B
HA-Null
HA-SMURF2
Flag-TBK1
Flag-S172A
Flag
HA
IP:
HA
HA
p-TBK1
Flag
β-actin
WCL
C
Flag-Null
Flag-TBK1
Flag-S172A
HA-SMURF2
HA
Flag
IP:
Flag
HA
Flag
p-TBK1
β-actin
WCL
D
1
148
344
748
SMURF2 (1-748Aa)
dC2 (148-748Aa)
dWW (1-147 and 344-748Aa)
dHECT (1-343Aa)
C2
WW
HECT
Flag-TBK1
Myc-Null
Myc-SMURF2
Myc-dC2
Myc-dWW
Myc-dHECT
Flag
Myc
IP:
Flag
Flag
Myc
GAPDH
WCL
E
Flag-Null
Flag-TBK1
Myc-Null
Myc-SMURF2
Myc-C716A
HA-Ub
HA
Flag
IP:
Flag
Flag
Myc
β-actin
WCL
F
Flag-Null
Flag-TBK1
Myc-SMURF2
Myc-dC2
Myc-dWW
Myc-dHECT
HA-Ub
HA
Flag
IP:
Flag
Flag
Myc
β-actin
WCL
G
Flag-Null
Flag-TBK1
Flag-S172A
SMURF2-WT
SMURF2-KO
HA-Ub
HA
Flag
IP:
Flag
Flag
SMURF2
β-actin
WCL
H
Flag-Null
Flag-S172A
Myc-Null
Myc-SMURF2
Myc-C716A
HA-Ub
HA
Flag
IP:
Flag
Flag
Myc
β-actin
WCL
I
Flag-Null
Flag-TBK1
Myc-Null
Myc-SMURF2
HA-Ub
HA-Ub K27R
HA-Ub K29R
HA
Flag
IP:
Flag
Flag
Myc
β-actin
WCL

J

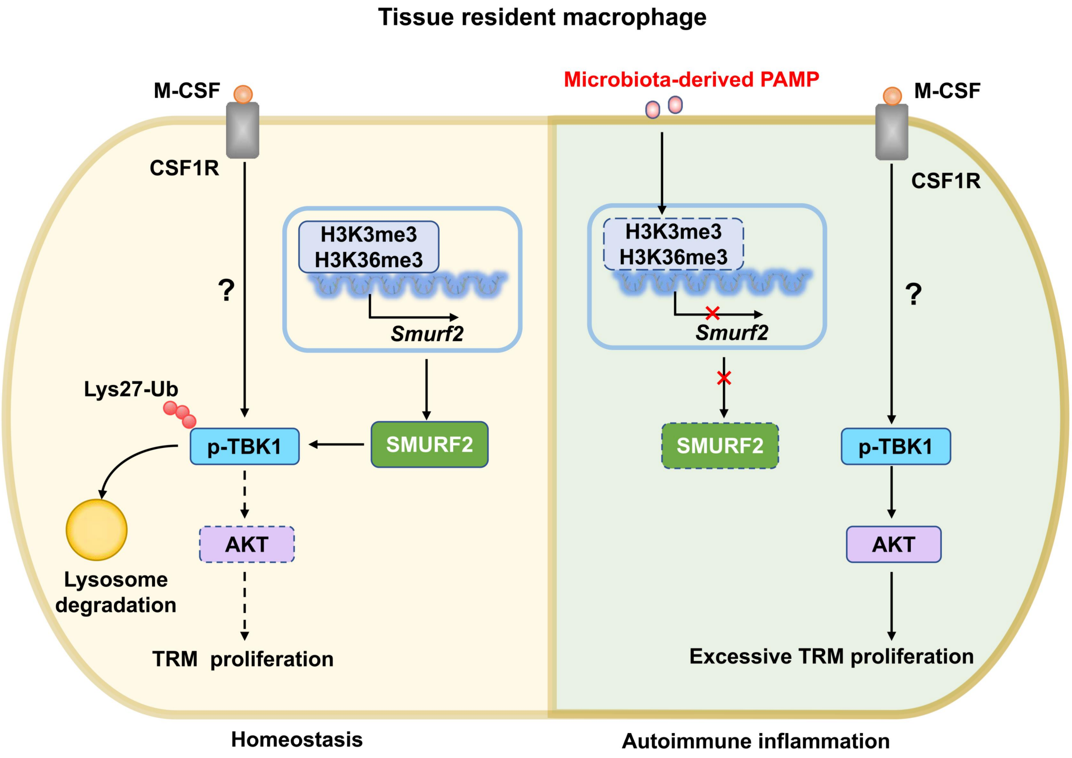

Figure S7. SMURF2 mediates ubiquitination and degradation of p-TBK1.

(A) Western blot analysis of p-TBK1 expression in M-CSF (100 ng/ml)-stimulated RAW264.7-Flag-Null (vector backbone) and RAW264.7-Flag-SMURF2 cells which were pretreated with MG132 (10 μM) or BFA (0.2 μM) for 6 h.

(B, C) Anti-HA **(B)** and anti-Flag **(C)** Co-IP of the interaction between HA-SMURF2 and Flag-TBK1 or Flag-TBK1 S172A in transfected HEK293T cells.

(D) Anti-Flag Co-IP of the interaction between Flag-TBK1 and Myc-SMURF2 or its trunked mutation Myc-dC2, Myc-dWW or Myc-dHECT in transfected HEK293T cells.

(E) Ubiquitination assay of Flag-TBK1 following immunoprecipitating TBK1 with anti-Flag antibody from lysates of HEK293T co-transfected with plasmids expressing Flag-TBK1 and HA-Ub with Myc-SMURF2 or Myc-SMURF2 C716A.

(F) Ubiquitination assay of Flag-TBK1 following immunoprecipitating TBK1 with anti-Flag antibody from lysates of HEK293T co-transfected with plasmids expressing Flag-TBK1 and HA-Ub with Myc-SMURF2, Myc-dC2, Myc-dWW or Myc-dHECT.

(G) Ubiquitination assay of Flag-TBK1 or Flag-TBK1-S172A in WT or CRISPR-Cas9-mediated SMURF2 knockout 293T (SMURF2 KO) cells.

(H) Ubiquitination assay of Flag-TBK1-S172A following immunoprecipitating S172A with anti-Flag antibody from lysates of HEK293T co-transfected with plasmids expressing Flag-TBK1-S172A, HA-Ub, Myc-SMURF2 or Myc-SMURF2 C716A.

(I) Ubiquitination assay of Flag-TBK1 following immunoprecipitating TBK1 with anti-Flag antibody from lysates of HEK293T cells co-transfected with plasmids expressing Flag-tagged TBK1, Myc-tagged SMURF2, and HA-tagged wild-type or K27R and K29R mutant Ub.

(J) A proposed model that E3 ligase SMURF2 restricts autoimmune diseases via targeting M-CSF-triggered TBK1/AKT signaling.

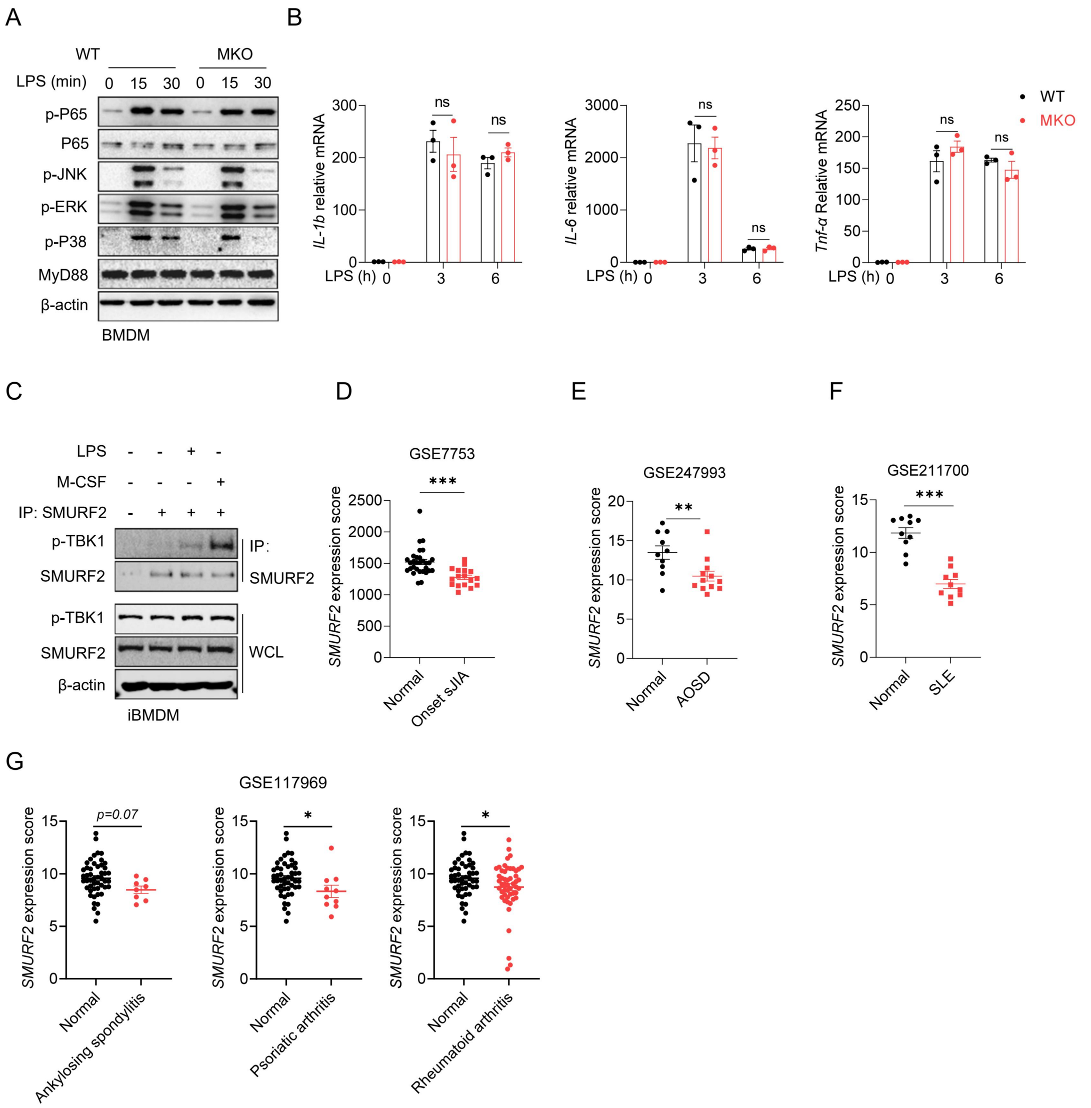

Figure S8. Decreased *SMURF2* expression in PBMC from patients with autoimmune diseases.

(A) Western blotting analysis of NF-kB/MAPK activation in WT and MKO BMDMs treated with LPS (100 ng/ml) for the indicated times.

(B) RT-qPCR analysis of *Il-6* and *Tnf-α* expression in WT and MKO BMDMs treated with LPS (100 ng/ml) for the indicated times.

(C) Anti-SMURF2 Co-IP of the interaction between SMURF2 and p-TBK1 in LPS (100 ng/ml, 15 min) or M-CSF (100 ng/ml, 30 min) treated iBMDMs.

(D-G) Relative *SMURF2* expression in the PBMC RNA-seq data of normal and Onset Systemic Juvenile Idiopathic Arthritis (Onset sJIA) **(D)**; Adult-onset Still's disease (AOSD) **(E)**; Systemic lupus erythematosus (SLE) **(F)**; Ankylosing spondylitis,

Psoriatic arthritis and Rheumatoid arthritis **(G)**.

Data are shown as mean ± SEM. Each dot represents a biological replicate (**D-G**) or a technical replicate **(B)**. Data are representative of at least two independent experiments **(A-C)**. *p<0.05, ** p<0.01, ***p<0.001, ns, Non-significant, P > 0.05. P values were calculated by using 2-tailed Student's t-tests (**B, D, E, F, G**).

	Normal<br>n=31	UC<br>n=36	CD<br>n=41
**Age(years)**			
<=30	0	9	13
30-50	9	11	11
=>50	22	16	17
**Gender**			
Male	20	17	22
Female	12	19	19
**Location**			
Colon	31	32	13
Terminal ileum	0	0	12
Rectum	0	4	4
Ileocecal	0	0	12

Table S1. Basic information of non-IBD normal control, UC, and CD patients from The First Affiliated Hospital, Zhejiang University School of Medicine (FAZHU). Human samples were obtained from FAZHU, including 31 non-IBD normal control human colon sections from the resection edges of tumor biopsies that appeared to be healthy at the histological level, and 36 human UC colon sections and 41 human CD colon sections from screening colonoscopies. The diagnosis of CD or UC was based on a standard combination of clinical, endoscopic, histological, and radiological criteria. The severity of macroscopic inflammation of the colon mucosa at colonoscopy was graded by a professional pathologist.

Gene	Sequence
mouse *β-actin*-F	AACAGTCCGCCTAGAAGCAC
mouse *β-actin*-R	CGTTGACATCCGTAAAGACC
mouse *Tnf-α*-F	CTGGGACAGTGACCTGGACT
mouse *Tnf-α*-R	GCACCTCAGGGAAGAGTCTG
mouse *Il-6*-F	AGTTGCCTTCTTGGGACTGA
mouse Il-6-R	TCCACGATTTCCCAGAGAAC
mouse *Smurf2*-F	ATGAAGTCATTCCCCAGCAC
mouse Smurf2-R	AACCGTGCTCGTCTCTCTTC
mouse *Il-17a*-F	TTTAACTCCCTTGGCGCAAAA
mouse Il-17a-R	CTTTCCCTCCGCATTGACAC
mouse *Csf1r*-F	AGGACCTGTTGGAGTTCCCTC
mouse *Csf1r*-R	TTTCGCCCTCACACTTGATGA
mouse *c-Myc*-F	ATGCCCCTCAACGTGAACTTC
mouse *c-Myc*-R	GTCGCAGATGAAATAGGGCTG
mouse *Ccnd1*-F	GCGTACCCTGACACCAATCTC
mouse *Ccnd1*-R	CTCCTCTTCGCACTTCTGCTC
human *SMURF2*-F	GGCAATGCCATTCTACAGATACT
human *SMURF2*-R	CCACTTTGGATCAAGCGTATTCT
human *GAPDH*-F	ATTCCACCCATGGCAAATTC
human *GAPDH*-R	GGATCTCGCTCCTGCAAGATG

Table S2. List of primers for qRT-PCR.

	Sequence
human *SMURF2*-gRNA-F	CACCGACTCCTCCAGACCTACCAGA
human *SMURF2*-gRNA-R	AAACTCTGGTAGGTCTGGAGGAGT
mouse TBK1-siRNA	CUGUGAAAGUGUAUGAGAAtt

Table S3. List of gRNAs and siRNA.

Reference:

[1] J. Liang, N. Wang, Y. Yao, Y. Wang, X. An, H. Wang, H. Liu, Y. Jiang, H. Li, X. Cheng, J. Xu, X. Liang, J. Lou, Z. Xin, T. Zhang, X. Wang, W. Lin, *The Journal of clinical investigation* **2024**.

[2] J. S. Park, F. S. Gazzaniga, M. Wu, A. K. Luthens, J. Gillis, W. Zheng, M. W. LaFleur, S. B. Johnson, G. Morad, E. M. Park, Y. Zhou, S. S. Watowich, J. A. Wargo, G. J. Freeman, D. L. Kasper, A. H. Sharpe, *Nature* **2023**, *617* (7960), 377.

[3] W. Lin, C. Ma, F. Su, Y. Jiang, R. Lai, T. Zhang, K. Sun, L. Fan, Z. Cai, Z. Li, H. Huang, J. Li, X. Wang, *Gut* **2017**, *66* (4), 597.

[4] H. Li, N. Wang, Y. Jiang, H. Wang, Z. Xin, H. An, H. Pan, W. Ma, T. Zhang, X. Wang, W. Lin, *EMBO reports* **2022**, *23* (11), e54603.

[5] H. Liu, W. Lin, Z. Liu, Y. Song, H. Cheng, H. An, X. Wang, *EMBO reports* **2021**, *22* (5), e52063.
